# Differential Expression of Non-Coding RNAs and Continuous Evolution of the X Chromosome in Testicular Transcriptome of Two Mouse Species

**DOI:** 10.1371/journal.pone.0017198

**Published:** 2011-02-14

**Authors:** David Homolka, Robert Ivanek, Jiri Forejt, Petr Jansa

**Affiliations:** Department of Mouse Molecular Genetics, Institute of Molecular Genetics, Academy of Sciences of the Czech Republic, Center for Applied Genomics, Prague, Czech Republic; University of Münster, Germany

## Abstract

**Background:**

Tight regulation of testicular gene expression is a prerequisite for male reproductive success, while differentiation of gene activity in spermatogenesis is important during speciation. Thus, comparison of testicular transcriptomes between closely related species can reveal unique regulatory patterns and shed light on evolutionary constraints separating the species.

**Methodology/Principal Findings:**

Here, we compared testicular transcriptomes of two closely related mouse species, *Mus musculus* and *Mus spretus*, which diverged more than one million years ago. We analyzed testicular expression using tiling arrays overlapping Chromosomes 2, X, Y and mitochondrial genome. An excess of differentially regulated non-coding RNAs was found on Chromosome 2 including the intronic antisense RNAs, intergenic RNAs and premature forms of Piwi-interacting RNAs (piRNAs). Moreover, striking difference was found in the expression of X-linked *G6pdx* gene, the parental gene of the autosomal retrogene *G6pd2*.

**Conclusions/Significance:**

The prevalence of non-coding RNAs among differentially expressed transcripts indicates their role in species-specific regulation of spermatogenesis. The postmeiotic expression of *G6pdx* in *Mus spretus* points towards the continuous evolution of X-chromosome silencing and provides an example of expression change accompanying the out-of-the X-chromosomal retroposition.

## Introduction

Spermatogenesis is a tightly regulated process of germ cell mitotic proliferation, differentiation, and two consecutive meiotic divisions ultimately forming spermatozoa. As testes contain various cell types including developing germ cells, the testicular transcriptome is complex with many yet uncharacterized transcripts. Beside the protein-coding genes, the testicular transcriptome is abundant in antisense transcripts [1], transcribed pseudogenes [2], retrogenes, and various types of non-coding RNAs including microRNAs (miRNAs) [3], and Piwi-interacting RNAs (piRNAs) [4], [5]. Several forms of these transcripts were shown to play a crucial role in spermatogenesis. Recently discovered piRNAs are specifically expressed in the male germ line and are involved in retrotransposon silencing [6]. Another class of small RNAs (microRNAs) was shown to escape inactivation of X chromosome in pachytene stage of spermatogenesis and was suggested to regulate this inactivation process [7], [8].

Differentiation of autosomal ancestors into the mammalian sex chromosomes started after the divergence of the mammalian and avian lineages and progressed during evolution [9]. The growing disparity of the X-Y pair obviously challenged performance of some vital processes such as control of gene dosage in somatic cells and proper execution of homologous recombination in testicular cells. In order to successfully complete male meiotic prophase, two processes have been evolving on sex chromosomes: suppression of recombination and transcriptional silencing – the latter process called meiotic sex chromosome inactivation (MSCI) [10], [11].

MSCI is established at mid-pachytene stage and then maintained to a substantial degree even postmeiotically [12], [13], although the multicopy X-linked genes were reported to escape this inactivation [14]. MSCI is believed to be the driving force for retroposition of spermatogenesis-related genes from X chromosome to autosomes [15], [16], [17]. Based on the comparative study in primates, the process of retroduplication has accelerated in recent evolution history and contributed significantly to the formation of new human genes functional in the male germline [18].

The genes involved in spermatogenesis play an important role during speciation, as demonstrated in *Drosophila* [19], [20], [21], [22] and in *Mus musculus* species [23]. Two studies compared transcriptomes of several tissues between human and chimpanzee [24] and between *Mus musculus* and *Mus spretus* mouse species [25]. Compared to other tissues, the testis revealed the highest expression divergence.

In this study we used mouse genomic tiling microarrays covering chromosomes X, Y, 2 and mitochondrial genome to study the differences in testicular transcriptome between *Mus musculus* and *Mus spretus*. As these mouse species diverged approximately 1.1–1.5 million years ago [26], their comparison could provide an insight into the recent evolutionary forces leading to different transcriptional activity during spermatogenesis. The tiling arrays enabled us to search for differentially expressed genomic regions regardless of their previous functional annotation.

Here we show differences in the expression pattern of the X-chromosome linked *G6pdx* gene, a parental gene of *G6pd2* recently retrotransposed on Chromosome 5. Our finding demonstrates the continuous evolution of the expression pattern of this parent-retrogene pair. Besides several known genes we observed differential expression of previously uncharacterized transcripts including antisense transcripts localized in introns of known genes and two piRNA clusters on Chromosome 2.

## Results

### Differential levels of testicular transcripts between *Mus spretus* and *Mus musculus*

As we wanted to determine how testicular transcriptomes differ between two mouse species by an unbiased experimental method we used mouse tiling arrays. We isolated RNA from testes of three males of *Mus spretus* (Spr) and *Mus musculus*, represented by C57BL/6J strain (B6). Only transcripts exceeding ∼200 nucleotides were hybridized to the chip. As the single-strand targets labeling was used and the probes on the chip come from the forward strand of the mouse genomic sequence, only reverse strand transcripts could be detected. This design enabled us to distinguish the antisense transcripts.

To reveal regions with differential expression between Spr and B6, we searched for genomic areas harboring the probes with diverged intensity ratios between Spr and B6. Briefly, the probes were ordered based on their individual chromosome coordinates and sliding windows of 100 probes were analyzed. The windows with at least 10% of probes exhibiting significantly different intensity between Spr and B6 (P<0.05) were merged to create the differentially regulated clusters (see Materials & Methods). Herefrom, the probes with significantly decreased intensity in Spr are stated as downregulated, the probes with increased intensity as upregulated. Counts of upregulated and downregulated probes are summarized in Table 1. Similarly, the clusters enriched in downregulated probes are called downregulated and vice versa.

**Table 1:** Summary of the unique probes present on the chip.

chromosome	probes	upregulated probes	% upregulated probes	downregulated probes	% downregulated probes
2	2750132	2696	0.10%	8952	0.33%
X	1600041	914	0.06%	2190	0.14%
Y	8826	4	0.05%	153	1.73%
Mitochondria	226	0	0.00%	102	45.13%

The probes with significantly decreased intensity in Spr (adjusted P<0.05) are listed as downregulated, the probes with increased intensity as upregulated.

Because of the sequence divergence between Spr and B6, the substantial number of downregulated probes and downregulated clusters might reflect this divergence and not the decreased expression. Indeed, we observed 11,397 downregulated probes compared to 3,614 upregulated probes in Spr. Moreover, the proportion of downregulated probes differed among the individual chromosomes. The highest proportion of downregulated probes was found in the mitochondrial genome. Almost 46% of mitochondrial probes were downregulated whereas none of them was upregulated. The downregulated probes were quite randomly distributed along the mitochondrial genome and created one downregulated cluster (Table 2 and Table S1). An abundance of downregulated probes was also a characteristic of Chromosome Y, with almost 35-fold excess of downregulated probes representing ∼1.8% of all probes on Chromosome Y.

**Table 2:** Summary of upregulated and downregulated clusters in *Mus spretus*.

chromosome	upregulated clusters	downregulated clusters
2	17	97
X	1	8
Y	0	3
Mitochondria	0	1

Differentially regulated clusters are regions enriched in either upregulated or downregulated probes.

**Figure 1:**
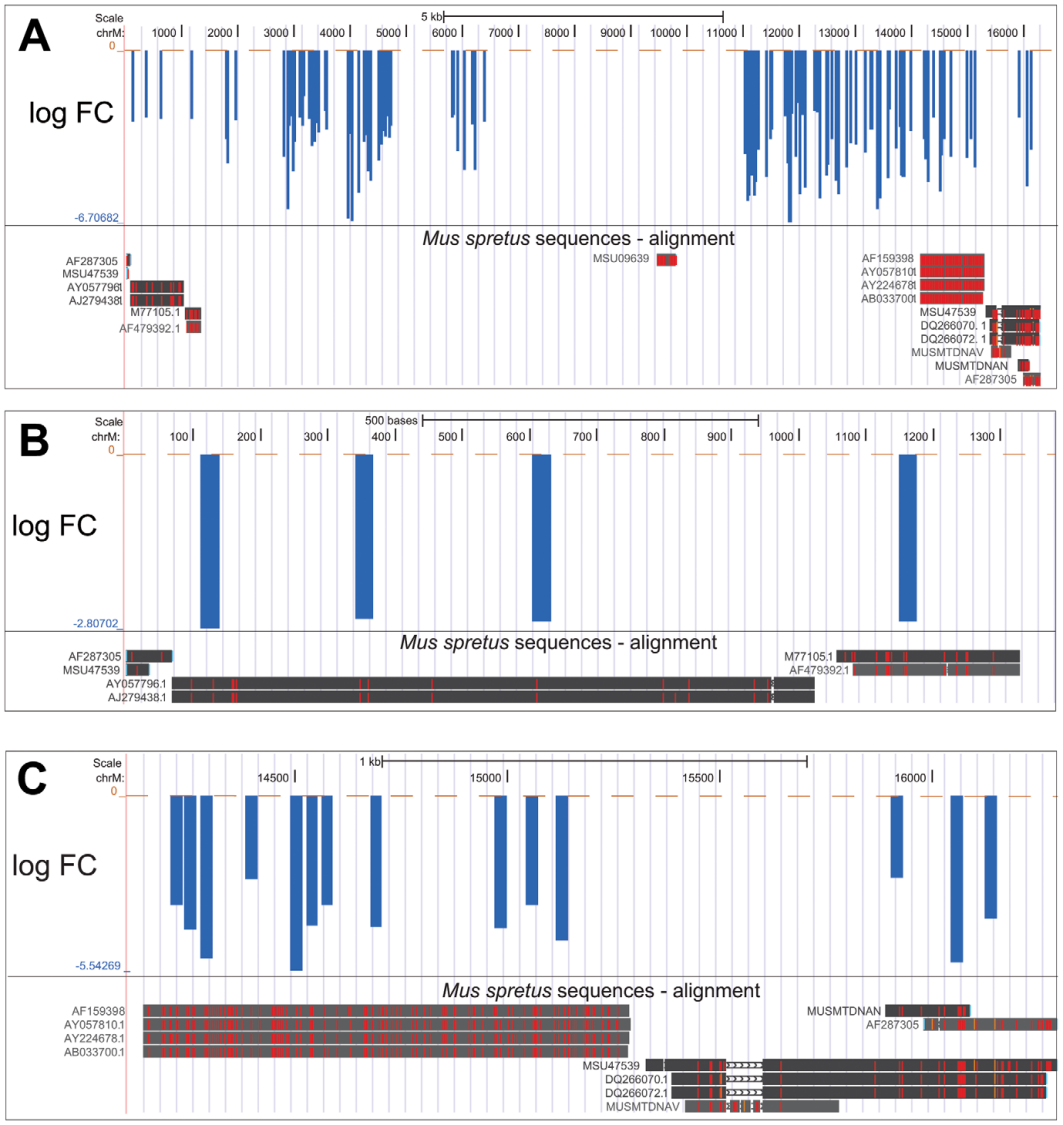
Downregulation of probes within the mitochondrial genome refers to sequence divergence. The individual probes are plotted along the mitochondrial genome coordinates as bars with the height corresponding to the log fold change difference in intensity between Spr and B6. (a) Two tracks are visualized: the upper one shows probes whose difference in intensity between Spr and B6 is significant at P<0.05. Below, the alignment of *Mus spretus* sequences obtained from GenBank (http://www.ncbi.nlm.nih.gov/Genbank/) to the B6 sequence is shown and positions of nucleotide differences are highlighted in red or orange. Red bars refer to SNPs and the orange color refers to insertion in the Spr sequence. Alignments are shown in detail in (b) and (c). The tracks were created using UCSC genome browser [50].

To verify the expected cause of overrepresentation of downregulated probes in the mitochondrial genome, we compared the mitochondrial sequences of Spr and B6. Fifteen *Mus spretus* mitochondrial DNA sequences obtained from GenBank (Table S2) covering ∼3680 nt exhibited ∼93.5% identity to the B6 sequence. Each of the 18 downregulated probes that mapped into this area, matched the sites of sequence divergence between Spr and B6 (Figure 1). It strongly suggests that the observed downregulation in Spr is due to the sequence divergence of the transcribed mitochondrial genomes between Spr and B6 and not due to the expression difference. We found 108 additional downregulated clusters on Chromosomes X, Y, and 2 (Table 2 and Table S1). Sequencing of these clusters would be necessary to distinguish between expression and sequence divergence.

### Downregulation of piRNA clusters on Chromosome 2 in Mus spretus

**Figure 2:**
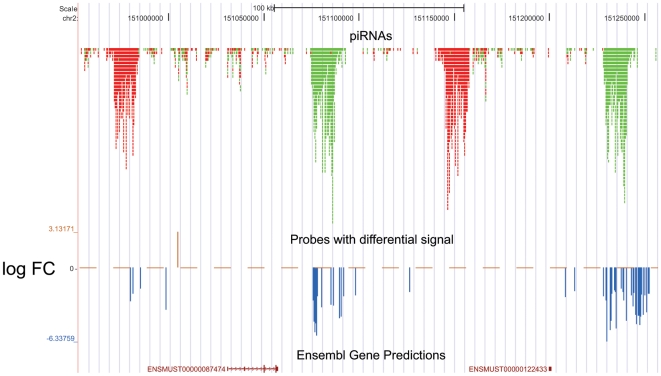
Downregulation of four piRNA clusters in chromosomal region chr2: 150,953,000–151,257,000. Three tracks are depicted. The upper one shows positions of piRNAs as obtained from piRNAbank (http://pirnabank.ibab.ac.in/). Reverse-strand piRNAs are shown in green, forward-strand piRNAs in red. Only the probes exhibiting significant differential intensity between Spr and B6 (P<0.05) are depicted in the middle track. Both clusters with reverse-strand piRNAs exhibit apparent abundance of downregulated probes. Predicted Ensemble transcripts are shown in the lowest track.

From the group of downregulated clusters in *Mus spretus* we focused only on two clusters of Chromosome 2 (Figure 2), since they are abundant in Piwi-interacting RNAs (piRNAs) with an obvious role in spermatogenesis [5], [6], [27].

Based on the piRNA sequences from piRNAbank (http://pirnabank.ibab.ac.in/) and published data [5], the region between chromosomal positions: 150,953,000-151,257,000 (UCSC build: mm9, NCBI build 37) consists of two piRNA clusters on forward genomic strand and two piRNA clusters on reverse genomic strand. All these clusters share high sequence similarity (>97%). The tiling arrays revealed an enrichment of downregulated probes in two reverse strand clusters in Spr (Figure 2). We assigned these differences to precursors of piRNAs rather than to their mature forms, because only the transcripts exceeding ∼200 nucleotides were assayed. Forward strand transcript clusters could not be detected due to the experimental design.

**Figure 3:**
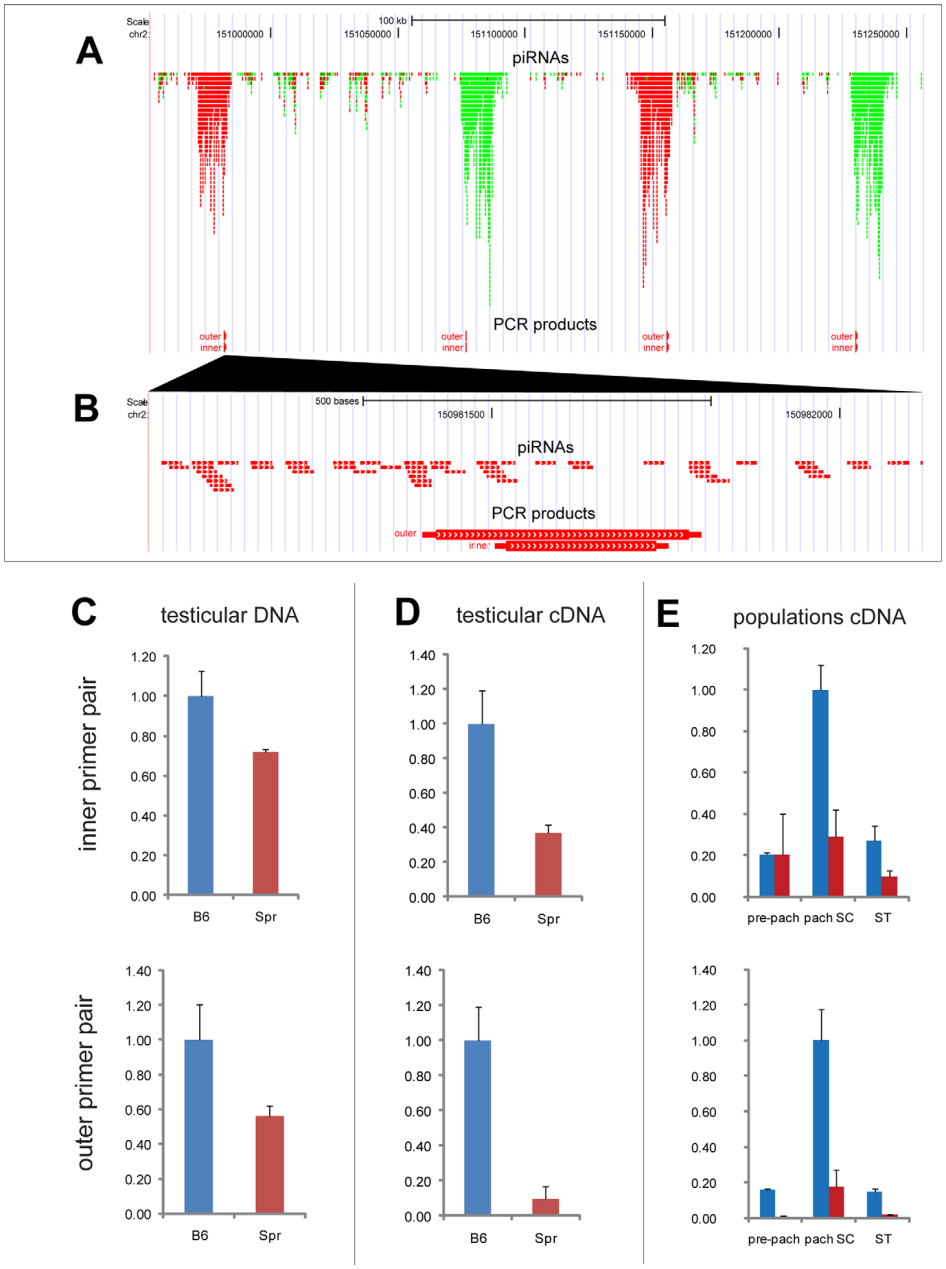
Quantitative real-time RT-PCR of piRNA clusters from Chromosome 2. (a) Two nested primer pairs were designed (outer and inner primer pairs), which amplify parts of all four piRNA clusters in chromosomal region chr2: 150,953,000–151,257,000. (b) Detailed view of part of the first cluster, which shows localization of the outer and inner primer PCR products. (c) Quantitative real-time PCR of testicular DNA indicate different copy number between Spr and B6. (d) Strong downregulation was observed in testicular cDNA. (e) In B6 the piRNA clusters were predominantly expressed in pachytene spermatocytes, with substantial downregulation in Spr. Two or three biological samples were used for each comparison. The expression was related to concentration and normalized to B6 expression. Standard deviations are plotted. Pre-pach: pre-pachytene spermatocytes; pach SC: pachytene spermatocytes; ST: spermatids.

In order to validate the tiling array results, we designed two nested primer pairs within the B6 sequence, which should amplify all four piRNA clusters (Figure 3a, 3b). Sequencing of the PCR products acquired from testicular genomic DNA confirmed amplification of all four clusters in B6, since unique polymorphisms for individual clusters were present (Figure S1). Even the Spr genomic DNA sequencing revealed several cases of polymorphisms but in different positions than in B6. The sequencing of the outer primer pair PCR product also confirmed sequence identity of the inner primer pair loci between Spr and B6 (Figure S1). As the real-time quantitative PCR (q-PCR) of genomic DNA provided lower amplification in Spr (1.4 and 1.8-fold for the inner and outer primer pair, respectively), we cannot exclude a possibility that less than four clusters are present in Spr genome (Figure 3c).

Using real-time quantitative Reverse Transcriptase-PCR (qRT-PCR) of testicular RNA, we confirmed the transcriptional decrease (2.7-fold for the inner primer pair and 10.5-fold for the outer primer pair) of the piRNA clusters in Spr (Figure 3d), which was substantially higher than the difference on the genomic DNA. The transcriptional decrease was also apparent when the Spr expression was compared to another laboratory strain C3H, which harbors all 4 clusters (Figures S1 and S2). To get insight into the transcriptional profile during spermatogenesis we performed qRT-PCR of RNA isolated from FACS-sorted populations (see Materials & Methods) of pre-pachytene and pachytene spermatocytes and spermatids. Using both pairs of primers we determined predominant expression of these piRNA clusters in pachytene spermatocytes of B6 mice and substantial downregulation in the same cell population of Spr species (Figure 3e). However, it remains to be elucidated whether the observed transcriptional downregulation is influenced by a possible lower number of piRNA clusters in the region.

**Figure 4:**
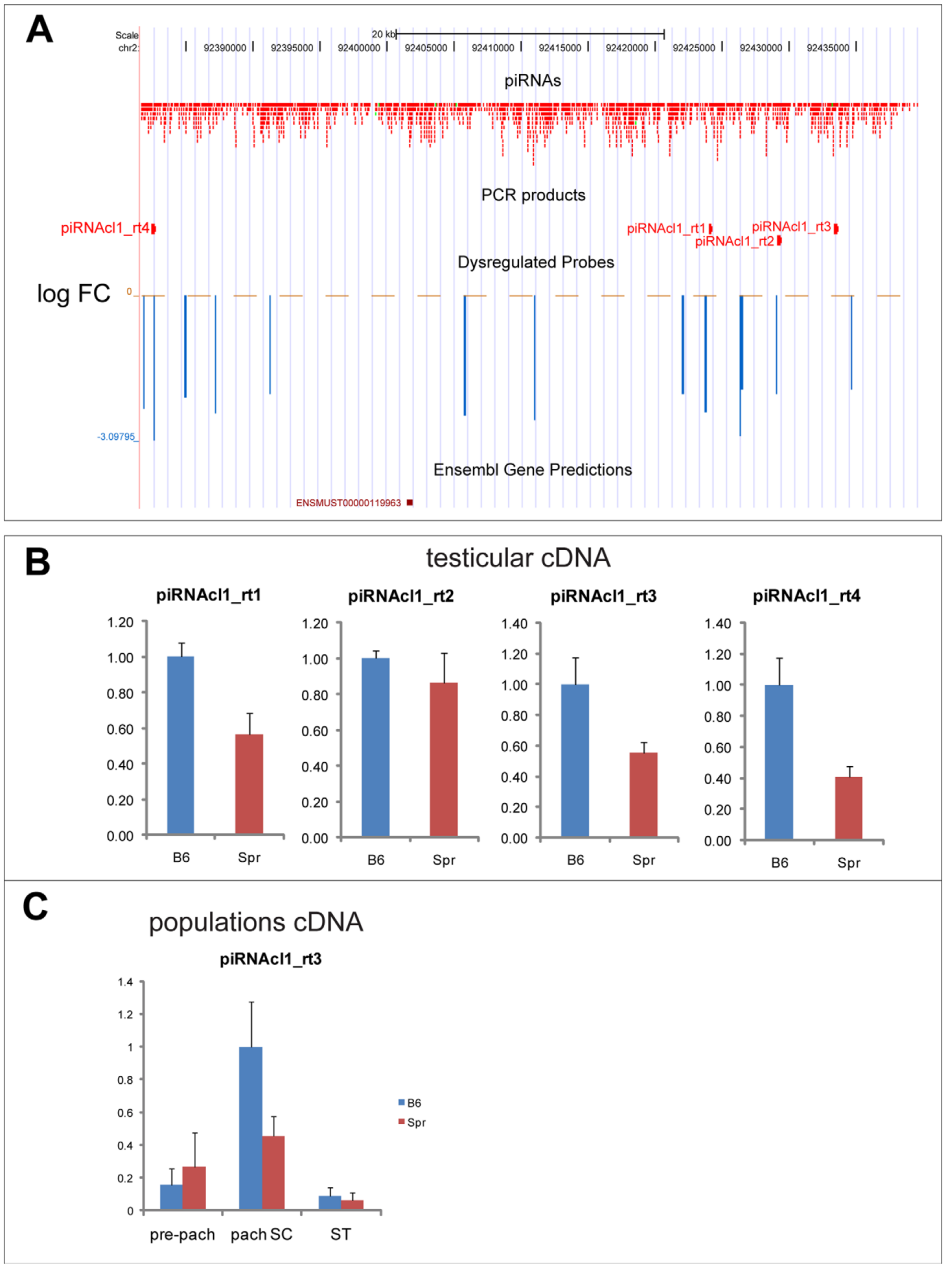
Downregulation of piRNA cluster in chromosomal region chr2: 92376642–92452541. (a) Four primers were designed to amplify piRNA precursors from the region to compare expression between Spr and B6. No downregulated cluster was found in this region on the tiling chip as this piRNA cluster corresponds to the genomic forward strand. (b) Still, the downregulation was discovered by qRT-PCR. (c) One primer pair was also used to investigate the expression pattern in spermatogenic populations, which confirmed the predominant expression in pachytene spermatocytes of B6 and downregulation in Spr. Three biological samples were used for each comparison. The expression was related to concentration and normalized to B6 expression. Standard deviations are plotted. Pre-pach: pre-pachytene spermatocytes; pach SC: pachytene spermatocytes; ST: spermatids.

Another large piRNA cluster on Chromosome 2 [5] corresponds to chromosomal positions 92,376,642–92,452,541 (UCSC build: mm9, NCBI build 37). Since this cluster contains only forward strand piRNAs, it is undetectable on the tiling array used. Hence, we designed four primer pairs to verify the expression of the piRNA precursors (Figure 4a). First, we performed the q-PCR on genomic DNA, which indicated the identical copy number of the piRNA region among the strains (Figure S2). Using qRT-PCR we detected significant transcriptional downregulation in Spr compared to B6 for three of four primers (Figure 4b). However, the expression of C3H was also lower and therefore similar to the expression of Spr (Figure S2). Therefore the diverged regulation of this piRNA region is rather the characteristic of B6 strain and does not discriminate between *Mus musculus* and *Mus spretus* species. Using one of the four primers we then confirmed the predominant expression of piRNA precursors in pachytene spermatocytes of B6 and substantial downregulation in the same cell population of Spr (Figure 4c).

### Abundance of upregulated non-coding RNAs on Chromosome 2

We identified 18 upregulated clusters in *Mus spretus* exclusively on the Chromosomes 2 and X (Table 3). Since the presence of upregulated probes can hardly be explained by sequence divergence, we consider the upregulated clusters as the regions with increased transcriptional activity. Still, the copy number variation might be involved.

**Table 3:** Summary of upregulated clusters in *Mus spretus*.

Mitochondria			
**no upregulated clusters**			

Chromosome Y			
**no upregulated clusters**			

Chromosome X				
nm	count of probes	start position	end position	annotation
1	221	71648841	71661093	G6pdx

Chromosome 2				
nm	count of Probes	start position	end position	annotation
1	100	10223083	10228173	intergenic
2	219	17264351	17272785	Nebl
3	452	48447801	48473250	intergenic
4	154	48474081	48480869	intergenic
5	111	58637714	58642857	antisense - intron of Upp2
6	289	62135015	62149145	antisense - intron of Slc4a10
7	199	91357783	91376827	antisense - intron of Ckap5
8	231	93358084	93368910	intergenic
9	205	103426984	103436926	antisense - intron of Abtb2
10	129	104565383	104573132	Depdc7
11	166	113131549	113143762	antisense - intron of Tmco5b
12	189	116138023	116146562	intergenic
13	108	117025053	117031232	intergenic
14	179	117516956	117529801	intergenic
15	190	126143678	126152270	Atp8b4
16	209	140482398	140490072	Flrt3
17	216	145836972	145853950	intergenic

The chromosomal positions of the listed clusters are displayed according to UCSC genome build mm9.

Seventeen upregulated clusters were found on Chromosome 2 (Table 3 and Figure S3). Four of the 17 clusters overlap the exons of known genes previously annotated to reverse strand. The genes involved are: *Nebl*, *Depdc7*, *Atp8b4* and *Flrt3*. Using qRT-PCR we verified the testicular expression of *Atp8b4* and *Flrt3* and confirmed their transcriptional upregulation in Spr, which was more than 4-fold for *Atp8b4* and 19-fold for *Flrt3*, respectively.

Strikingly, the majority of the upregulated clusters (13/17) do not correspond to any annotated transcript, suggesting the existence of novel transcripts in Spr. According to the annotation of RefSeq (http://www.ncbi.nlm.nih.gov/RefSeq/) and Ensemble (www.ensembl.org), these clusters can be divided into two groups: the first group contains five upregulated clusters, which reside in the introns of known forward strand genes and therefore represent possible new antisense transcripts. The second group consists of eight clusters that do not match any known gene, thus occupying intergenic regions.

**Figure 5:**
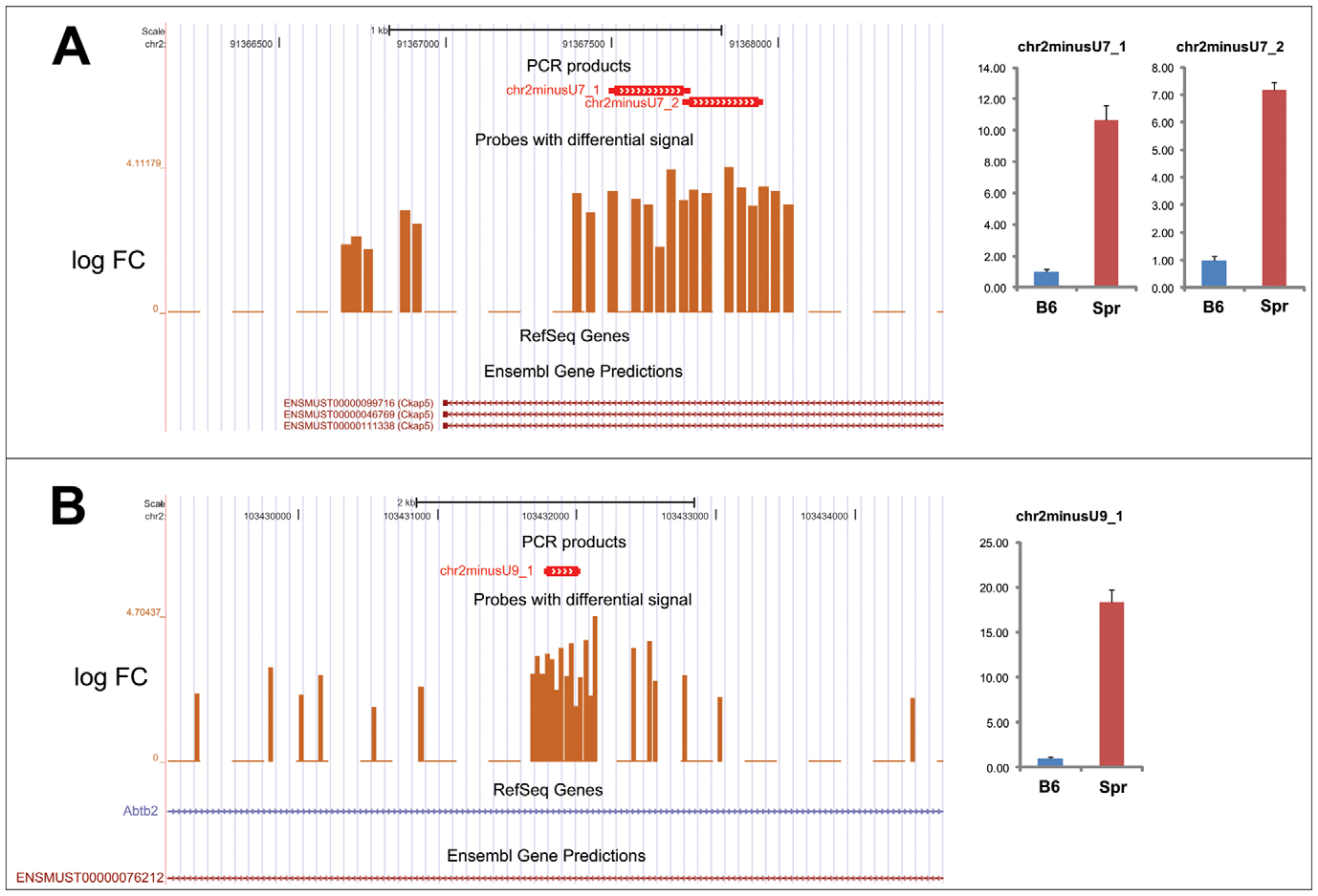
Upregulation of non-coding antisense RNAs. Two upregulated clusters from chromosome 2 are depicted: (a) cluster 7, which is an antisense to the *Ckap* gene, and (b) cluster 9, which is an antisense to the *Abtb2* gene. Differentially regulated probes are depicted together with the positions of the PCR products, which were quantified using qRT-PCR to verify the upregulation in testicular germ cell suspension. Three biological samples were used for each comparison. The expression was related to concentration and normalized to B6 expression. Standard deviations are plotted.

**Figure 6:**
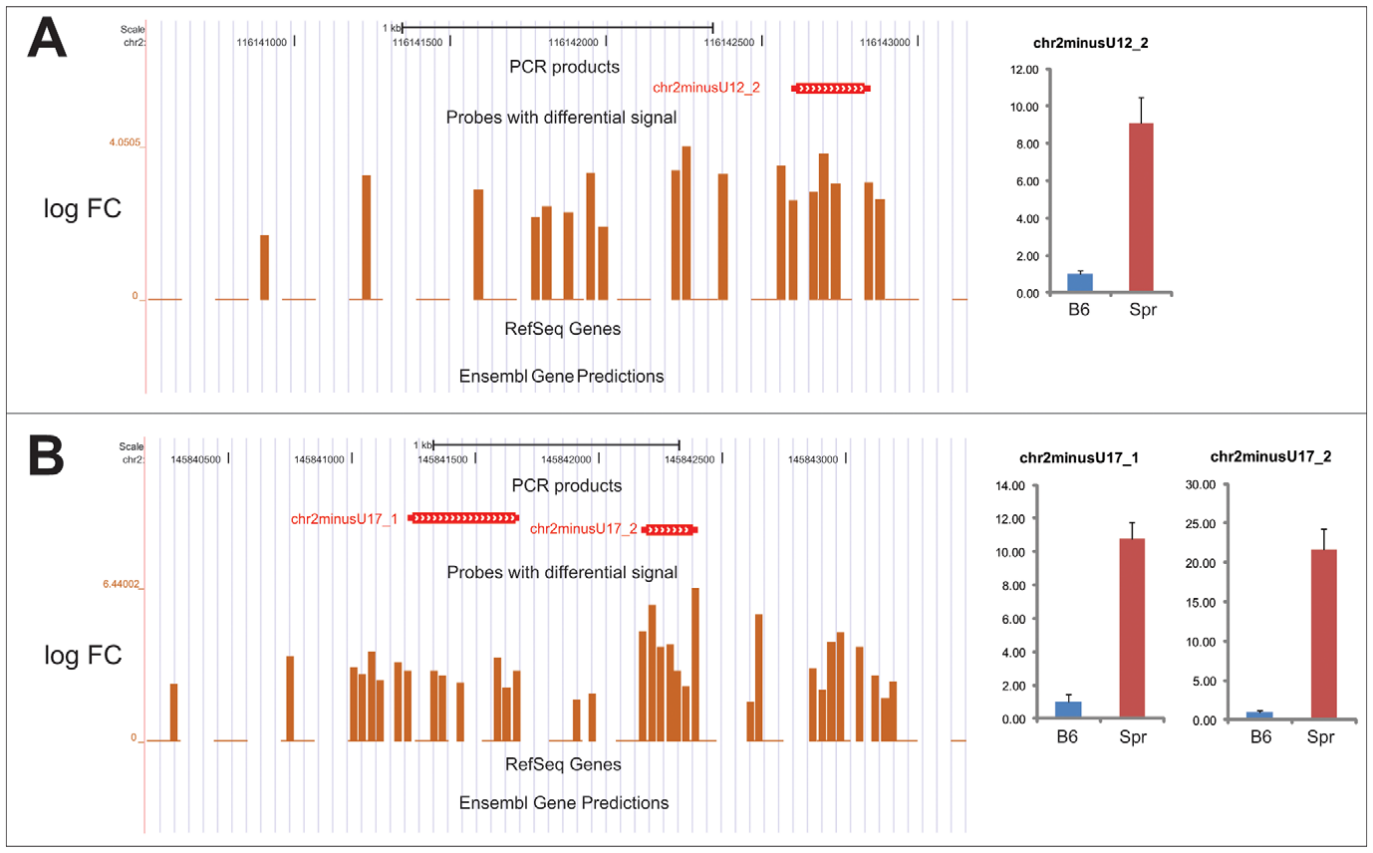
Upregulation of non-coding intergenic RNAs. Two upregulated clusters from chromosome 2 are depicted: (a) cluster 12 and (b) cluster 17, which lie in the intergenic regions. Differentially regulated probes are depicted together with positions of the PCR products, which were quantified using qRT-PCR to verify the upregulation in testicular germ cell suspension. Three biological samples were used for each comparison. The expression was related to concentration and normalized to B6 expression. Standard deviations are plotted.

We verified the transcriptional upregulation of several of these clusters (4 of 13) using qRT-PCR. Clusters 7 and 9 represent possible antisense transcripts, which reside in introns of the *Ckap5* or *Abtb2* gene, respectively. The expression in Spr strongly exceeded the B6 expression: more than 10-fold for cluster 7 (Figure 5a) and 18-fold for cluster 9 (Figure 5b). Clusters 12 and 17 are potential new transcripts with strong upregulation. More than 9-fold upregulation was detected for cluster 12 (Figure 6b) and 10-fold for cluster 17 (Figure 6b).

### *G6pdx* escapes postmeiotic silencing in Mus spretus

**Figure 7:**
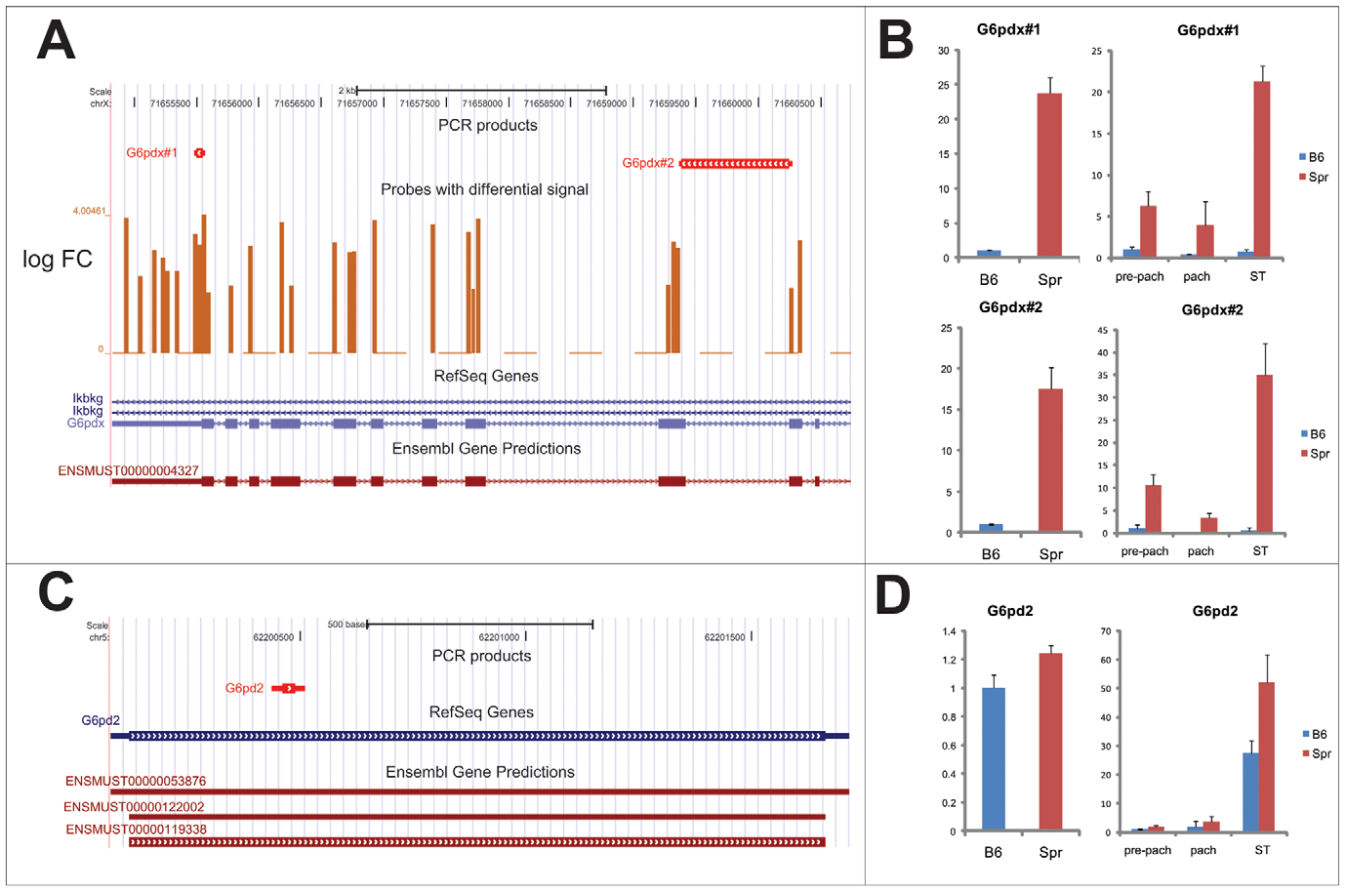
*G6pdx* escapes postmeiotic silencing in *Mus spretus*. (a) Two pairs of primers were designed to verify expression differences of *G6pdx* between Spr and B6. (b) Both pairs of primers confirmed upregulation in testicular single-cell suspension. While *G6pdx* is meiotically and postmeiotically silenced in B6, predominant spermatid expression was discovered in Spr. (c) One pair of primers was designed for *G6pdx* retrogene *G6pd2*, which is localized on chromosome 5. *G6pd2* was predominantly postmeiotically expressed in both B6 and Spr. (d) Two or three biological samples were used for each comparison. The expression was related to concentration and normalized to B6 expression. Standard deviations are plotted. Pre-pach: pre-pachytene spermatocytes; pach: pachytene spermatocytes; ST: spermatids.

We found a single upregulated cluster on the X chromosome in Spr (Figure 3). It overlaps the exons of the *G6pdx* gene, which encodes glucose-6-phosphate dehydrogenase and exhibits both meiotic and postmeiotic transcriptional silencing in the mouse [28]. In order to verify its upregulation in Spretus we performed qRT-PCR with two pairs of primers (Gpdx#1, G6pdx#2) (Figure 7a). The primers match exon 13 and encompass exons 4 and 5, respectively. Partial sequencing of *Mus* s*pretus G6pdx* cDNA overlapping exons 6 to 13 confirmed the absence of SNPs in G6pdx#1 primers (data not shown). Both pairs of primers used in qRT-PCR exhibited extensive transcriptional upregulation of *G6pdx* in the testis of Spr (>15 times) (Figure 7b). Remarkably different expression profiles between Spr and B6 were determined in isolated spermatogenic populations (Figure 7b). In strong contrast to low gene expression in all the B6 germ cell populations, the Spr *G6pdx* exhibited strong preferential expression in spermatids, exceeding the B6 expression more than 25 times. The upregulation was detected even in the populations of pachytene spermatocytes. However, as these isolated populations contained a proportion of spermatids (<5%), we cannot rule out the possibility that the observed upregulation was due to contaminating cells.

The meiotic and postmeiotic silencing of *G6pdx* in B6 is compensated by expression of its functional retrogene *G6pd2* on Chromosome 5 [28]. To verify whether G6pdx primers amplify exclusively *G6pdx* and not its retrogene, we performed qPCR on genomic DNA of Spr and B6 using G6pdx#2 primers (Figure S4). Both B6 and Spr samples contained a single PCR amplicon of size ∼907 nt, which corresponds to the length of the genomic *G6pdx* region spanning the intron 4. Remarkably, no other amplicons, especially the intronless *G6pd2* (81nt), were detected. The obtained PCR products were sequenced and confirmed to match the intron 4 of *G6pdx*. Moreover, we performed q-PCR on genomic DNA of Spr and B6 and achieved similar relative levels of amplification, showing that *G6pdx* in Spr is a single-copy gene as it is in B6 (Figure S4).

To find out whether the transcriptional dysregulation of *G6pdx* in Spr is accompanied by different expression of its retrogene, we measured the expression of *G6pd2* in the whole testis and in spermatogenic populations by qRT-PCR (Figure 7c). Comparison between Spr and B6 showed a similar pattern of this retrogene expression and demonstrated the preferential spermatid expression in both mouse species (Figure 7d). We verified the match of the *G6pd2* primers by sequencing the corresponding parts of the *G6pd2* gene in both Spr and B6 (data not shown).

**Figure 8:**
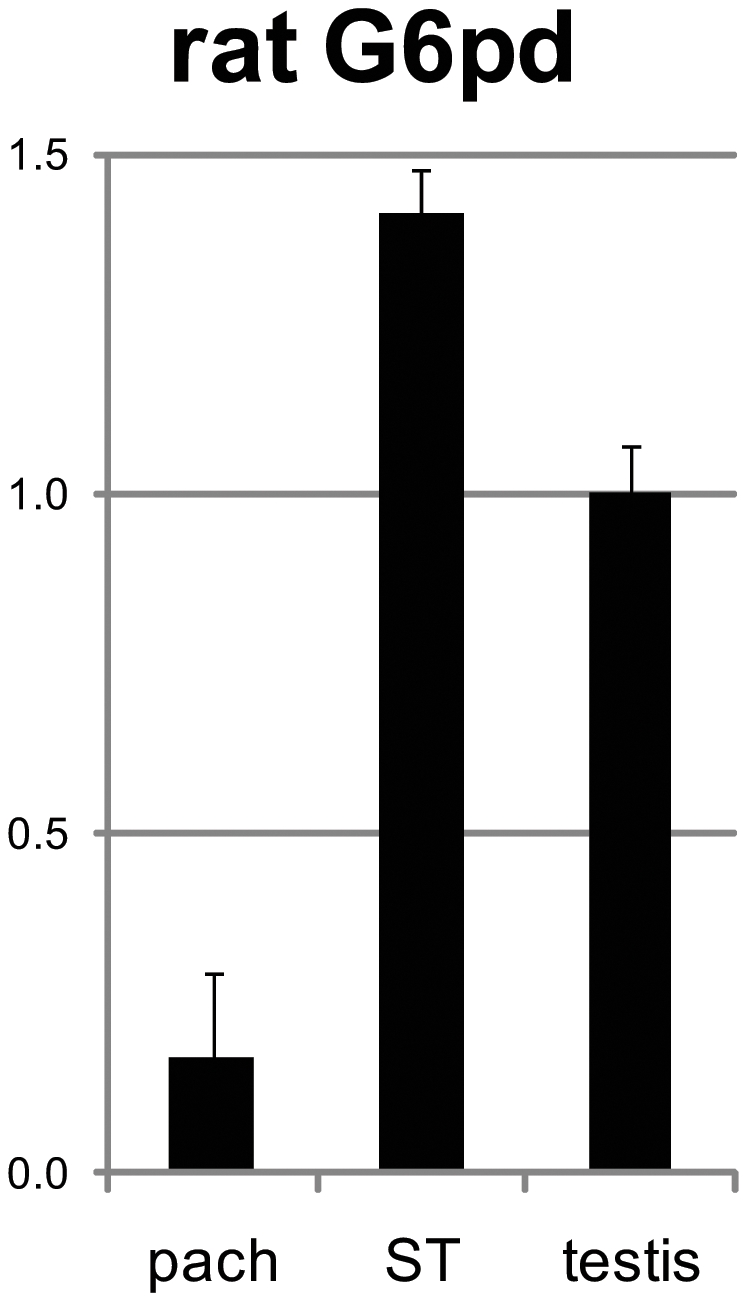
Rat X-chromosomal *G6pd* is postmeiotically expressed in spermatogenesis. Quantitative reverse transcriptasePCR (qRT-PCR) was performed on total RNA from testicular germ cell suspension (testis) and sorted germ cell populations of pachytene spermatocytes (pach) and spermatids (ST). *G6pd* is silenced in pachytene spermatocytes and reactivated postmeiotically in spermatids. Two to four replicas were used for each cell type and the expression was normalized to the expression in testicular germ cells. Standard deviations are plotted.

To further elucidate the ancestral state of *G6pdx* expression, we explored the expression profile of the X-linked *G6pd* gene in rat spermatogenesis. The qRT-PCR of total RNA from rat testicular germ cells and from sorted germ cells, namely pachytene spermatocytes and spermatids, showed that the rat X-chromosomal *G6pd* gene was silenced in pachytene spermatocytes and reactivated postmeiotically in spermatids (Figure 8).

## Discussion

The rate of sequence variation between *Mus* s*pretus* and laboratory strains is estimated as one variant every 50 base pairs [29] or 130 base pairs [30] which might account for part of the downregulated probes detected by tiling arrays. The bias in favor of downregulated probes was most apparent for the mitochondrial genome and Y chromosome in concordance with the expected higher differentiation rate of mitochondrial DNA and Chromosome Y loci [31]. The identified SNPs in positions of downregulated probes suggested that the observed downregulation of Spr mitochondrial genome is caused by sequence divergence.

Thus, the observed downregulated clusters in Spr might either reflect lower level of transcription or higher sequence divergence of the transcribed regions. We focused here on two such regions on Chromosome 2, which harbor “pachytene piRNAs”, whose expression start in mid-late pachytene spermatocytes and persist until spermatids [4], [5], [32]. Their role is unclear as opposed to “pre-pachytene piRNAs”, which are involved in transposon silencing [6]. A possible function of pachytene piRNAs in translational regulation was suggested based on their time-dependent differential association with polysomes and ribonucleotide particles [32], [33].

We demonstrated that the piRNAs of these two regions are transcribed as long precursors (pre-piRNAs), in agreement with the proposed model of individual piRNA clusters as single transcription units. This model is based on the profound strand asymmetry of pachytene piRNA clusters [4] flanked by inverted transposable elements [34].

The finding of almost exclusive expression of the pre-piRNAs in pachytene spermatocytes was unexpected, as it is in contrast with the postmeiotic abundance of their mature forms [4]. Possible explanations are that transcriptional regulation of these piRNAs of Chromosome 2 differs from pachytene piRNAs of other chromosomes, or that mature piRNAs, in contrast to their precursors, persist in spermatids from prior stages of spermatogenesis.

Moreover, using qRT-PCR we observed decreased expression of the pre-piRNAs in Spr, which originated from selected parts of both studied regions. Comparison to C3H, another laboratory strain representing *Mus musculus* species, confirmed the downregulation for one of the piRNA regions, indicating a species-specific regulation. However, more independent representatives from both species would be necessary to exclude intraspecific variation in piRNA regulation. Besides, we cannot entirely exclude the contribution of different genome architecture between the species, especially the copy number variation.

Out of 17 upregulated clusters on Chromosome 2 in Spr, 13 are not annotated to exons of known genes and are presumably long non-coding RNAs (>200 nt in length; lncRNAs). These transcripts either reside in introns of known genes in antisense orientation (5 clusters) or lie in intergenic regions (8 clusters). High complexity of mammalian transcriptome, comprising loads of lncRNAs [1], [35], [36], [37], [38], [39], was described earlier in both human and mouse using high-throughput and high-resolution techniques such as tiling arrays and RNA-seq. A regulatory function was suggested for some of these ncRNAs based on their tissue specific expression [37], [40], [41]. The overrepresentation of lncRNAs among upregulated transcripts in Spr reflects their substantial contribution to the testicular transcriptome divergence between species, and indicates their functionality in species-specific spermatogenesis regulation. Furthermore lncRNAs are likely involved in epigenetic regulation of chromatin state [42] and their general role in control of regulatory networks of differentiation and development was suggested [43].

Consequently, the acquired interspecific differences in non-coding RNA transcriptome of spermatogenic cells could also contribute to species specific barriers and might be involved in molecular pathways leading to male hybrid sterility phenomenon [44]. To assess profile of the whole genome non-coding RNA transcriptome and the overall expression of mature piRNAs along the whole piRNA clusters, deep RNA sequencing would be necessary, which would also enable characterization of the sequence divergence at a time.

The inactivation of X chromosome during spermatogenesis, known as MSCI [11] is the proposed driving force of retroposition of X-chromosome genes to autosomes [15], [17], [45] and might pose limits to the X-chromosome expression divergence. Indeed, we found the only upregulated cluster on Chromosome X in Spr, which mapped to the *G6pdx* gene, compared to 17 single-copy upregulated clusters on Chromosome 2. As only the probes with one perfect match to the genome were analyzed, possible differences in the expression of multi-copy genes on the X chromosome were outside the scope of this study. Retrogenes are supposed to functionally substitute their X-linked transcriptionally silenced parental genes. Indeed, a number of these retrogenes, including *G6pd2* retrogene of *G6pdx* [28], [45], [46], were shown to be expressed during meiosis while their parental genes were silenced.

Our observation of differential spermatogenic expression profile of *G6pdx* between two mouse species represented by Spr and B6 illustrates such a retroposition. Move of the *G6pd* retro-copy from X to Chromosome 5 was an evolutionarily recent event, because the functional retrogene *G6pd2* exists only in the mouse [28]. In B6, the expression profiles of *G6pdx* and *G6pd2* conform to functional substitution of the X-chromosomal parental gene by its autosomal counterpart: *G6pdx* is expressed premeiotically, whereas *G6pd2* is expressed in spermatids. In Spr we found a strikingly different pattern of expression with *G6pdx* transcribed also in spermatids. As the *G6pd2* expression profile is similar in B6 and Spr, it should be capable of functional substitution of *G6pdx* in both species. We suggest the following order of events to explain the observed divergence of *Mus musculus* and *Mus* s*pretus* species: *G6pdx* was retroposed and *G6pd2* obtained its spermatid-specific expression pattern in the common ancestor of both species. After *Mus musculus* and *Mus* s*pretus* diverged, the *G6pdx* became post-meiotically silenced in the *Mus musculus* lineage, while its expression in spermatids of *Mus spretus*, though functionally redundant, was still maintained. The concept of the *G6pdx* postmeiotic expression as an ancestral state is supported by our observation of postmeiotic expression of the X-chromosomal *G6pd* gene in the rat.

The different regulation of *G6pdx* also points out the distinct extent of postmeiotic silencing of the X chromosome between the species. Although mainly multicopy genes on X chromosome escape postmeiotic sex chromosome inactivation (PMSCI) [14], here we show that even a single-copy gene might escape the inactivation in a species-specific manner. Thus, the different postmeiotic activity of *G6pdx* between *Mus spretus* and *Mus musculus* emphasizes the continuous evolution of X-chromosome silencing following the successful retroposition.

In summary, *Mus spretus* and *Mus musculus* show divergence in the male germ cell expression repertoire of non-coding RNAs and in the extent of X-chromosome (post) meiotic silencing, both of which might contribute to their reproductive isolation.

## Materials and Methods

### Animals

The C57BL/6J inbred strain was obtained from the Jackson Laboratory in 1998 (Bar Harbor, ME), the C3H/HeJ substrain (C3Sn.BLiA-Pde6b+/DnJ) was obtained from Yann Hérault in 2007 (ICS, France). Since then both strains were maintained in the Specific Pathogen-Free barrier facility of the Institute of Molecular Genetics, AS CR. The males of *Mus spretus* originated from wild-derived animals caught in Montferrier-sur-Lez, Languedog (France). They underwent inbreeding by successive brother – sister mating to generation 9 by J. Pialek and were obtained from the Institute of Vertebrate Biology (Brno, Czech Republic). All male mice used in the present experiments were two months old.

The three months old male of Brown-Norway BN.lx rat strain was a gift from M. Pravenec and P. Mlejnek (Institute of Physiology, AS CR).

Animal experiments were conducted based on Animal Use Protocols approved by the Institutional Animal Care and Use Committee (IACUC) of the Institute of Molecular Genetics, Prague, (Protocol-project number: 87/2006). The principles of laboratory animal care observed the Czech Republic Act for Experimental Work with Animals (Decree No. 207/2004 Sb., and Acts Nos. 246/92 Sb. and 77/2004 Sb.) fully compatible with the corresponding EU regulations and standards, namely Council Directive 806/609/EEC and Appendix A of the Council of Europe Convention ETS123.

### FACS characterization and isolation of spermatogenic populations

Populations of mouse pre-pachytene spermatocytes, pachytene spermatocytes, and spermatids and rat pachytene spermatocytes, and spermatids were isolated using fluorescence-activated cell sorting according to ref. [47] with minor modifications as described earlier [48]. Briefly, spermatogenic tubules of mice or rats euthanized by cervical dislocation were incubated in enriched Krebs-Ringer bicarbonate medium (EKRB) with collagenase (0.5 mg/ml; Sigma) for 20 min at 32°C on a shaker. The tubules were separated by a cell strainer (BD Falcon) and incubated with collagenase under the same conditions. The suspension was filtered by the cell strainer and the cells washed twice by EKRB containing 1% FCS. Finally, the cells were diluted in 1 ml of EKRB with 1% FCS and stained with Hoechst 33342 (13 µg/ml) for 1 hour at 32°C. Propidium iodide was added just before FACS analysis to concentration 2 µg/ml. Individual populations were sorted according to red and blue Hoechst emission (Bastos et al. 2005) directly into the RLT buffer Plus of the AllPrep DNA/RNA Micro Kit (Qiagen). Aliquots were sorted to EKRB medium for subsequent immunofluorescense analysis. Based on SCP3, SCP1 and γH2AX immunostaining and morphology of the cells, we determined the composition of FACS-isolated populations. The fraction of mouse pre-pachytene spermatocytes consisted of leptotene and zygotene spermatocytes (∼60%), and early pachytene spermatocytes (∼40%). The population of both mouse and rat pachytene spermatocytes displayed ∼90% of the cells at the late pachytene stage. The population of mouse and rat spermatids consisted of ∼90% of the spermatids.

### RNA isolation

RNA of the spermatogenic populations was isolated using AllPrep DNA/RNA Micro Kit (Qiagen).

For the isolation of testicular RNA, the single-cell testicular suspension was prepared: spermatogenic tubules of mice or rat euthanized by cervical dislocation were incubated in enriched Krebs-Ringer bicarbonate medium (EKRB) with collagenase (0.5 mg/ml; Sigma) for 20 min at 32°C on a shaker. The tubules were filtrated with cell strainer (BD Falcon) and incubated with collagenase under the same conditions. The suspension was filtered and the cells washed twice by EKRB containing 1% FCS. Finally, the cells were resuspended in 600 µl of RLT buffer with 1% β-mercaptoethanol and RNA was isolated using RNeasy Mini isolation kit (Qiagen).

### DNA isolation

DNA was isolated from B6, C3H and Spr testes, from which the tunica was removed, and from rat spleen. The tissue samples were homogenized in 5 ml Fasano A (200 mM NaCl, 20 mM EDTA, 20 mM Tris: pH = 8, 0.5% SDS) solution using a glass Wheaton homogenizer. After addition of Fasano B (0.5% SDS) and proteinase K (to concentration 0.05 mg/ml) the samples were incubated overnight at 50°C on a shaker. The DNA was then extracted by one round of phenol-chloroform-isoamylalcohol extraction and two rounds of chloroform-isoamylalcohol extraction. Finally, the genomic DNA was precipitated by ethanol and wound up onto an inoculation loop. After washing with 70% ethanol, it was dried and diluted in TE.

### Tiling array analysis

Affymetrix GeneChip® Mouse Tiling 2.0R B Array was used in order to investigate differences in transcriptional activity between *Mus spretus* (Spr) and *Mus musculus* species represented by C57BL/6J strain (B6). The Tiling 2.0R B Array covers around 14% of the mouse genome involving Chromosomes X, Y, 2 and mitochondrial genome. Two µg of total testicular RNA was isolated from three independent individuals of each species and used to prepare biotinylated single-stranded target using the Affymetrix protocol (Whole Transcript (WT) Double-Stranded Target Assay Manual, 702179 Rev. 3, Affymetrix) with a slight modification: the synthesis of second-strand cDNA was omitted in the final step. Therefore, only cDNA of reverse strand transcripts was hybridized to the chips.

Sequences of the 25 nt long probes were aligned to the chromosomal positions (UCSC genome build mm9) using MUMmer (http://mummer.sourceforge.net/). Only probes with one perfect match and without any near-match (match of 23 or 24 nt) were left. We also filtered out the probes arising from the reverse strand and/or matching other chromosomes than X, Y, 2 or mitochondrial genome. Intensities of the remaining probes were quantile-normalized using R (http://www.r-project.org/). LogFC values of Spr versus B6 comparison together with P values were calculated for individual probes using limma package [49] from Bioconductor (www.bioconductor.org). The P values were adjusted using Benjamini & Hochberg correction. To find out the dysregulated clusters between Spr and B6 we constructed sliding windows of 100 probes, which were moved along the chromosomal coordinates. For each window step the proportion of significantly upregulated or downregulated probes (adjusted P<0.05) was calculated. Overlapping sliding windows with at least 10% of significantly dysregulated probes were merged to create the dysregulated clusters. The results were visualized in the UCSC browser [50].

The dataset is MIAME compliant and is deposited in the NCBI Gene Expression Omnibus (GEO) with series accession number GSE19633.

### Real-time quantitative Reverse Transcriptase-PCR (qRT-PCR) and quantitative real-time PCR (q-PCR) of genomic DNA

Total RNA isolated from the sorted spermatogenic populations or testicular single-cell suspension was reverse-transcribed using Mu-MLV Reverse Transcriptase (Invitrogen).

Quantitative real-time PCR of cDNA or genomic DNA was performed with Light-Cycler DNA Fast Start Master SYBR green I kit (Roche) in a Light Cycler 2000 machine at Tm = 61°C. RT minus reactions were negative. Amplification values were calculated as (1/(2^∧^Cp))/input, where Cp is the crossing point value and input was the amount of DNA or reverse transcribed RNA used as a template. The sequences of primers used are listed in the Table 4.

**Table 4:** Summary of oligonucleotide primers used in qRT-PCR and qPCR.

primer	direction	sequence (5′ ->3′)
outer	Forward	ATGTGGACAAACATACAGTAG
outer	reverse	TTCCCACAGAATATCCCT
inner	forward	TGTGGCTGTCTGCATC
inner	reverse	GAAAATGGTGCCTGGAG
piRNAcl1_rt1	forward	AGTGGGCTAGTGATGC
piRNAcl1_rt1	reverse	TCAGAAACCAGCTAAGTGT
piRNAcl1_rt2	forward	TCTCAATGGGCATGGTT
piRNAcl1_rt2	reverse	GCACTAATAATAATAGCGCCT
piRNAcl1_rt3	forward	CAGTCAAAGCGCCTCC
piRNAcl1_rt3	reverse	CTGTCTCGACACTAAAGC
piRNAcl1_rt4	forward	ACCGTCCAAATAGGGC
piRNAcl1_rt4	reverse	GTCAGACCATAACAATCAGTG
chr2minusU7_1	forward	CGTCCACTACCTGTGAT
chr2minusU7_1	reverse	CATAGCAATTACCACGACT
chr2minusU7_2	forward	CAAAGTCGTGGTAATTGCT
chr2minusU7_2	reverse	TCCTATCTGGGCCAAG
chr2minusU9_1	forward	GCAGGTTTTAGTGAGAGC
chr2minusU9_1	reverse	GGTCCCTAATACGGTCG
chr2minusU12_2	forward	CAGGGTGCAATTCCAGA
chr2minusU12_2	reverse	TGCAGGAGAGACATAAAGT
chr2minusU17_1	forward	CAACTCATTATCGTTAGTGCC
chr2minusU17_1	reverse	CCTGGGTAAGAGGTGT
chr2minusU17_2	forward	GTCCACATTCATCTCTAGGT
chr2minusU17_2	reverse	GCCATTCACATTTGGAGT
G6pdx#1	forward	AGTGGGTGAACCCTCACAA
G6pdx#1	reverse	AAAAGGGAAGATGCAGAAAGG
G6pdx#2	forward	GAAAGCAGAGTGAGCCCTTC
G6pdx#2	reverse	CATAGGAATTACGGGCAAAGA
G6pd2	forward	CTCCTATGTAGTTGGCCAGTATGA
G6pd2	reverse	TGGTGCAGGGCATTAATGTAG
ratG6pd	forward	GTGCAAGCGTAACGAG
ratG6pd	reverse	CCAGGATGAGGCGTTC

## Supporting Information

Table S1 A table summarizing all *Mus spretus* downregulated clusters.
(PDF)

Table S2 A table summarizing GenBank mitochondrial sequences of *Mus* s*pretus* that were used for sequence comparison to B6.
(PDF)

Figure S1 DNA sequence alignment of clusters from piRNA region: chr2:150,953,000-151,257,000.
(PDF)

Figure S2 Quantitative real-time PCRs on genomic DNA and testicular cDNA for piRNA regions on Chromosome 2. Comparisons between Spr, B6 and C3H.
(PDF)

Figure S3 Summary of *Mus spretus* upregulated clusters on chromosome 2.
(PDF)

Figure S4 Copy number evaluation of *G6pdx* in Spretus by quantitative real-time PCR.
(PDF)
