# Supplementary material for: Differential Expression of Non-Coding RNAs and Continuous Evolution of the X Chromosome in Testicular Transcriptome of Two Mouse Species
Source: PLoS One. 2011 Feb 14;6(2):e17198. doi: 10.1371/journal.pone.0017198 (PMC3038937; doi:10.1371/journal.pone.0017198)
Supplement: Table S1 — A table summarizing all Mus spretus downregulated clusters. (PDF) [file pone.0017198.s001.pdf]

Table S1. Summary of all *Mus spretus* downregulated clusters. Positions refer to nucleotide positions according to UCSC build mm9.

Mitochondria

#	count of Probes	start position	end position
1	226	5	16269

Chromosome Y

#	count of Probes	start position	end position
1	124	59932	74194
2	314	1788406	2783673
3	220	2793797	2862069

Chromosome X

#	count of Probes	start position	end position
1	150	35548698	35554321
2	276	43600161	43622671
3	181	72947107	72973073
4	443	120080980	121223287
5	391	121279522	121327359
6	156	123344817	123351631
7	164	131002259	131010055
8	141	140440265	140470276

chromosome 2

#	count of Probes	start position	end position
1	223	3329545	3341060
2	282	4050309	4066579
3	154	4542080	4549230
4	315	5791496	5807446
5	120	11745881	11764182
6	239	13966351	13980552
7	126	13985400	13996338
8	239	21108138	21122016
9	179	22434369	22450293
10	221	25464127	25473370
11	198	25778884	25789542
12	175	28547965	28555242
13	195	30029045	30041239
14	176	32133404	32144923
15	190	32236742	32244938
16	107	32989328	32993255
17	155	38578709	38584852
18	146	39262811	39286217
19	122	47212456	47230109
20	173	48766947	48783141
21	115	50148387	50154312
22	296	51773083	51790216
23	213	52547290	52562750
24	193	56976358	56985169
25	172	56994038	57005429
26	206	59745452	59753773
27	148	65919843	65926907
28	229	66514916	66527949
29	199	66534149	66543849
30	202	68083632	68091805
31	265	68379710	68406941
32	279	74134021	74146905
33	208	74547847	74556727
34	215	74988072	74996095
35	200	75809898	75821196
36	163	79168405	79179465
37	100	82824700	82828333
38	238	84864491	84877577
39	545	90288233	90315914
40	199	90503997	90518008
41	193	92368659	92384370
42	186	93795242	93803512

43	131	93820381	93825650
44	124	93835902	93841846
45	191	93842049	93850366
46	152	103601092	103607551
47	541	103825128	103869908
48	138	104626105	104634592
49	111	109885603	109891118
50	108	110890709	110897924
51	276	110971947	110985605
52	213	111022695	111034886
53	103	111061524	111068612
54	160	111141859	111150289
55	156	118064636	118075967
56	212	120276517	120289065
57	188	120557024	120565189
58	163	121210440	121225511
59	148	121226830	121240373
60	191	124912138	124921627
61	332	124922289	124941475
62	181	127000442	127030089
63	135	130366233	130372987
64	100	131125371	131129517
65	128	132664715	132671629
66	161	134419704	134426087
67	199	134988258	135002900
68	158	135009242	135016064
69	135	135036531	135043240
70	170	136277935	136294226
71	173	136332354	136339653
72	293	136906568	136918153
73	124	140088737	140095173
74	181	140594217	140602815
75	166	146065526	146071663
76	195	150827617	150836592
77	190	151048706	151177274
78	305	151204205	151312321
79	197	151566860	151584918
80	146	153285107	153294647
81	258	155987644	156002740
82	181	156517805	156526959
83	199	156825512	156834798
84	162	164409204	164417731
85	123	166722958	166732028
86	293	168014379	168029173
87	188	168082145	168093551
88	220	168900538	168913316
89	182	170169380	170176935
90	223	170287534	170299252
91	229	170594480	170606763
92	207	171762917	171774024
93	227	174103114	174112034
94	144	178089195	178094816
95	210	178128259	178138974
96	193	179381652	179390052
97	187	180413882	180422022
