# Supplementary material for: Differential Expression of Non-Coding RNAs and Continuous Evolution of the X Chromosome in Testicular Transcriptome of Two Mouse Species
Source: PLoS One. 2011 Feb 14;6(2):e17198. doi: 10.1371/journal.pone.0017198 (PMC3038937; doi:10.1371/journal.pone.0017198)
Supplement: Table S2 — A table summarizing GenBank mitochondrial sequences of Mus spretus that were used for sequence comparison to B6. (PDF) [file pone.0017198.s002.pdf]

Table S2. Summary of GenBank mitochondrial sequences of *Mus spretus*, which were used for sequence comparison to B6.

accession	description	leght of alignment	identity to B6
AB033700.1	cytb gene	1140	91.60%
AY224678.1	cytb gene	1145	91.30%
AY057810.1	cytb gene	1145	91.70%
AF159398.1	cytb gene	1144	91.50%
U47539.1 (MSU47539)	DNA type 1 control region	971	94.50%
DQ266072.1	trnP gene, D loop	885	95.20%
DQ266070.1	trnP gene, D loop	885	95.20%
M77119.1 (MUSMTDNAV)	DNA fragment	359	94.40%
M77111.1 (MUSMTDNAN)	DNA fragment	198	93.50%
AF287305.1	tRNA-Phe gene	322	87.50%
M77105.1 (MUSMTDNAH)	DNA fragment	272	93.80%
AF479392.1	16S rRNA	247	94.00%
AJ279438.1	12S rRNA	957	98.30%
AY057796.1	12S rRNA	957	98.40%
U09639.1 (MSU09639)	NADH dehydrogenase subunit 3	335	91.70%

Alignments were made and the identity calculated using BLAT (Kent 2002).

Kent, W. J. (2002). "BLAT--the BLAST-like alignment tool." Genome Res **12**(4): 656-64.
