# Supplementary material for: Differential Expression of Non-Coding RNAs and Continuous Evolution of the X Chromosome in Testicular Transcriptome of Two Mouse Species
Source: PLoS One. 2011 Feb 14;6(2):e17198. doi: 10.1371/journal.pone.0017198 (PMC3038937; doi:10.1371/journal.pone.0017198)
Supplement: Figure S1 — DNA sequence alignment of clusters from piRNA region: chr2:150,953,000-151,257,000. (PDF) [file pone.0017198.s003.pdf]

Figure S1

```
                 ....|....| ....|....| ....|....| ....|....| ....|....| ....|....|
                     125        135        145        155        165        175
B6_cl2A          TGTGGACAAA CATACAGTAG TTTGTATACC TTCTGCACTA AACAGAATCC TTTTGACCAG
B6_cl2B_rc       TGTGGACAAA CATACAGTAG TTTGTATACC TTCTGCACTA AACAGAATCC TTTTGACCAG
B6_cl2C          TGTGGACAAA CATACAGTAG TTTGTATACC TTCTGCACTA AACAGAATCC TTTTGACCAG
B6_cl2D_rc       TGTGGACAAA CATACAGTAG TTTGTATACC TTCTGCACTA AACAGAATCC TTTTGACCAG
B6_DNA_seq       ---------- ---------- ---------- ---------- -------TCC TTTTGACCAG
C3H_DNA_seq      ---------- ---------- ---------- ---------- -------TCC TTTTGACCAG
Spr_DNA_seq      ---------- ---------- ---------- ---------- ---------C TTTTGACCAG
Clustal Consens                                                       * **********

                 ....|....| ....|....| ....|....| ....|....| ....|....| ....|....|
                     185        195        205        215        225        235
B6_cl2A          GATGGAGGAG GCACACAGCT GCCTGGCCTT GGTTAAGCTT TCTACTGTGG CTGTCTGCAT
B6_cl2B_rc       GATGGAGGAG GCACACAGCT GCCTGGCCTT GGTTAAGCTT TCTACTGTGG CTGTCTGCAT
B6_cl2C          GATGGAGGAG GCACACAGCT GCCTGGCCTT GGTTAAGCTT TCTACTGTGG CTGTCTGCAT
B6_cl2D_rc       GATGGAGGAG GCACACAGCT GCCTGGCCTT GGTTAAGCTT TCTACTGTGG CTGTCTGCAT
B6_DNA_seq       GATGGAGGAG GCACACAGCT GCCTGGCCTT GGTTAAGCTT TCTACTGTGG CTGTCTGCAT
C3H_DNA_seq      GATGGAGGAG GCACACAGCT GCCTGGCCTT GGTTAAGCTT TCTACTGTGG CTGTCTGCAT
Spr_DNA_seq      GATGGAGGAG GCACACAGYT GCCTGGCCTT GGTTAWGCTT TCTACTGTGG CTGTCTGCAT
Clustal Consens  ********** ******** * ********** ***** **** ********** **********

                 ....|....| ....|....| ....|....| ....|....| ....|....| ....|....|
                     245        255        265        275        285        295
B6_cl2A          CCATACCAGG TTTGTCAGCC TGTGGGGATC CCACCTGCTC CCTTGGCATT GGAGAGCAAC
B6_cl2B_rc       CCATACCAGG TTTGTCAGCC TGTGGGGAGC CCACCTGCTC CCGTGGCATT GGAGAGCAAC
B6_cl2C          CCATACCAGG TTTGTCAGCC TGTGGGGAGC CCACCTGCTC CCGTGGTATT GGAGAGCAAC
B6_cl2D_rc       CCATACCAGG TTTGTCAGCC TGTGGGGAGC CCACCTGCTC CCTTGGCATT GGAGAGCAAC
B6_DNA_seq       CCATACCAGG TTTGTCAGCC TGTGGGGAKC CCACCTGCTC CCKTGGYATT GGAGAGCAAC
C3H_DNA_seq      CCATACCAGG TTTGTCAGCC TGTGGGGAGC CCACCTGCTC CCKTGGYATT GGAGAGCAAC
Spr_DNA_seq      CCATACCAGG --TGTCAKCC TGTGGGGAGC CCAGCTGCTC CCTTGGCATT GGAGAGCAGC
Clustal Consens  **********   ***** ** ******** * *** ****** ** *** *** ******** *

                 ....|....| ....|....| ....|....| ....|....| ....|....| ....|....|
                     305        315        325        335        345        355
B6_cl2A          ATGACATGGG GAAGGGACTT CAGGTGACAT TGAAATATGG GTAATTAATC TTTGACCATC
B6_cl2B_rc       ATGACATGGG GAAGGGACTT CAGGTGACAT TGAAATATGG GTAATTAATC TTTGACCATC
B6_cl2C          ATGACATGGG GAAGGGACTT CAGGTGACAT TGAAATATGG GTAATTAATC TTTGACCATC
B6_cl2D_rc       ATGACATGGG GAAGAGACTT CAGGTGACAT TGAAATATGG GTAATTAATC TTTGACCATC
B6_DNA_seq       ATGACATGGG GAAGRGACTT CAGGTGACAT TGAAATATGG GTAATTAATC TTTGACCATC
C3H_DNA_seq      ATGACATGGG GAAGGGACTT CAGGTGACAT TGAAATATGG GTAATTAATC TTTGACCATC
Spr_DNA_seq      ATGGCWTGGG GAAGGGACTT CAGGTGACAT TGAAATATGG GTAATTAATC TTTGACCATC
Clustal Consens  *** * **** **** ***** ********** ********** ********** **********

                 ....|....| ....|....| ....|....| ....|....| ....|....| ....|....|
                     365        375        385        395        405        415
B6_cl2A          CAGGAAGGCC ATGTGTTAAG ATGAATCTCT CACTCATTCC TCCTGAGTGA CCTTTTTCCC
B6_cl2B_rc       CAGGAAGGCC ATGTGTTAAA ATGAATCTCT CGCTCATTCC TCCTGAGTGA CCGTTTTTCC
B6_cl2C          CAGGAAGGCC ATGTGTTAAA ATGAATCTCT CGCTCATTCC TCCTGAGTGA CCGTTTTTCC
B6_cl2D_rc       CAGGAAGGCC ATGTGTTAAA ATGAATCTCT CGCTCATTCC TCCTGAGTGA CGGTTTTTGC
B6_DNA_seq       CAGGAAGGCC ATGTGTTAAR ATGAATCTCT CRCTCATTCC TCCTGAGTGA CSKTTTTYSC
C3H_DNA_seq      CAGGAAGGCC ATGTGTTAAA ATGAATCTCT CGCTCATTCC TCCTGAGTGA CSKTTTTYSC
Spr_DNA_seq      CAGGAAGGCC ATGTGTTAAA ATGAATCTCT CSCTCATTCC TCCTGAGTGA CCCTTTTTCC
Clustal Consens  ********** *********  ********** * ******** ********** *  ****  *

                 ....|....| ....|....| ....|....| ....|....| ....|....| ....|....|
                     425        435        445        455        465        475
B6_cl2A          CCACATCATC AGCCCTCCTT TCCCATTAAT TCTTGGCCTC CAGGCACCAT TTTCTATTGG
B6_cl2B_rc       CCCCATCATC AGCCCTCCTT TCCCATTAAT TCTTGGCCTC CAGGCACCAT TTTCTATTGG
B6_cl2C          CCCCATCATC AGCCCTCCTT TCCCATTAAT TCTTGGCCTC CAGGCACCAT TTTCTATTGG
B6_cl2D_rc       CCCCATCATC AGCCCTCCTT TCCCATTAAT TCTTGGCCTC CAGGCACCAT TTTCTATTGG
B6_DNA_seq       CCMCATCATC AGCCCTCCTT TCCCATTAAT TCTTGGCCTC CAGGCACCAT TTTCTATTGG
C3H_DNA_seq      CCCCATCATC AGCCCTCCTT TCCCATTAAT TCTTGGCCTC CAGGCACCAT TTTCTATTGG
Spr_DNA_seq      CCCCATCATC AGCCCTCCTT TACCATTAAT TCTTGGCCTC CAGGCACCAT TTTCTATTGG
Clustal Consens  ** ******* ********** * ******** ********** ********** **********

                 ....|....| ....|....| ....|....| ....|....| ....|....| ....|....|
                     485        495        505        515        525        535
B6_cl2A          ATAGTCTCAT T-AACTTTCT CAGAAGGGAT ATTCTGTGGG AATTGCTTTC TTAGATGACA
B6_cl2B_rc       ATAGTCTCAT TCAACTTTCT CAGAAGGGAT ATTCTGTGGG AATTGCTTTC TTAGATGACA
B6_cl2C          ATAGTCTCAT TCAACTTTCT CAGAAGGGAT ATTCTGTGGG AATTGCTTTC TTAGATGACA
B6_cl2D_rc       ATAGTCTCAT TCAACTTTCT CAGAAGGGAT ATTCTGTGGG AATTGCTTTC TTAGATGACA
B6_DNA_seq       ATAGTCTCAT T--------- ---------- ---------- ---------- ----------
C3H_DNA_seq      ATAGTCTCAT T-C------- ---------- ---------- ---------- ----------
Spr_DNA_seq      ATAGTCTCAT TCATCTTTCT TAGAAGGGA- ---------- ---------- ----------
Clustal Consens  ********** *
```

Figure S1. Sequencing of clusters from piRNA region: chr2:150,953,000-151,257,000

The outer primer pair was used to amplify genomic testicular DNA from Spr and B6. PCR products were sequenced and obtained consensus sequences were aligned to the sequences of individual B6 piRNA clusters (A,B,C,D), which were obtained from UCSC genome build mm9. Sequencing of the PCR products confirmed the amplification of all four B6 piRNA clusters as confirmed by presence of mixture of nucleotides (shown in yellow) in positions of polymorphisms, which distinguish the individual clusters (in grey). The C3H sequence was practically identical to B6 sequence, although in some polymorphic positions we were able to identify only peak for one of the two nucleotides probably to lower quality of the sequence. Also in Spr the mix of sequences was obtained, but in different positions. Positions of polymorphisms between Spr clusters are shown in green. Sequence identity was confirmed between Spr, C3H and B6 for the inner primer pair, whose positions are visualized in blue.
