# Supplementary material for: Differential Expression of Non-Coding RNAs and Continuous Evolution of the X Chromosome in Testicular Transcriptome of Two Mouse Species
Source: PLoS One. 2011 Feb 14;6(2):e17198. doi: 10.1371/journal.pone.0017198 (PMC3038937; doi:10.1371/journal.pone.0017198)
Supplement: Figure S2 — Quantitative real-time PCRs on genomic DNA and testicular cDNA for piRNA regions on Chromosome 2. Comparisons between Spr, B6 and C3H. (PDF) [file pone.0017198.s004.pdf]

Figure S2

Quantitative real-time PCR of piRNA clusters in chromosomal region chr2: 150,953,000-151,257,000

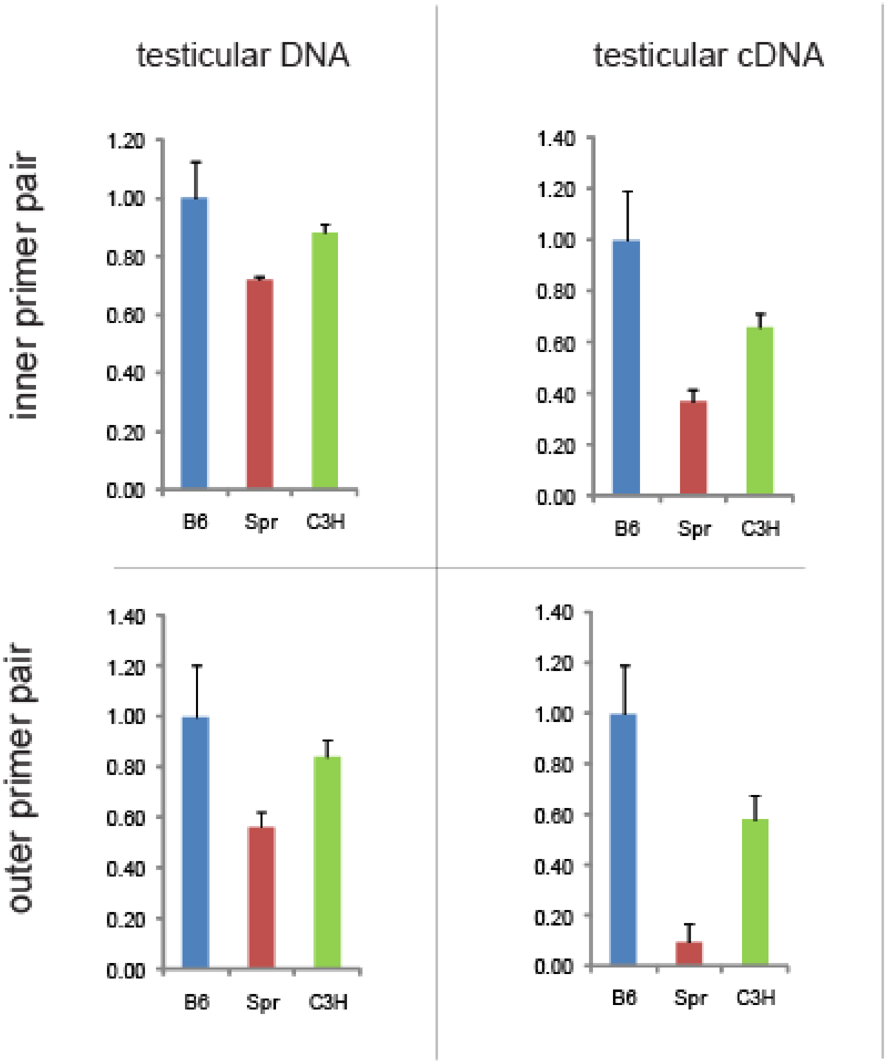

Two nested primer pairs were designed (outer and inner primer pairs), which amplify parts of all four piRNA clusters in chromosomal region chr2: 150,953,000-151,257,000. Comparison between Spr and B6 shows downregulation on testicular cDNA in Spr, which exceeds the differences on testicular DNA. The downregulation in Spr is also apparent when related to C3H.

Quantitative real-time PCR of piRNA clusters in chromosomal region chr2: 92376642-92452541

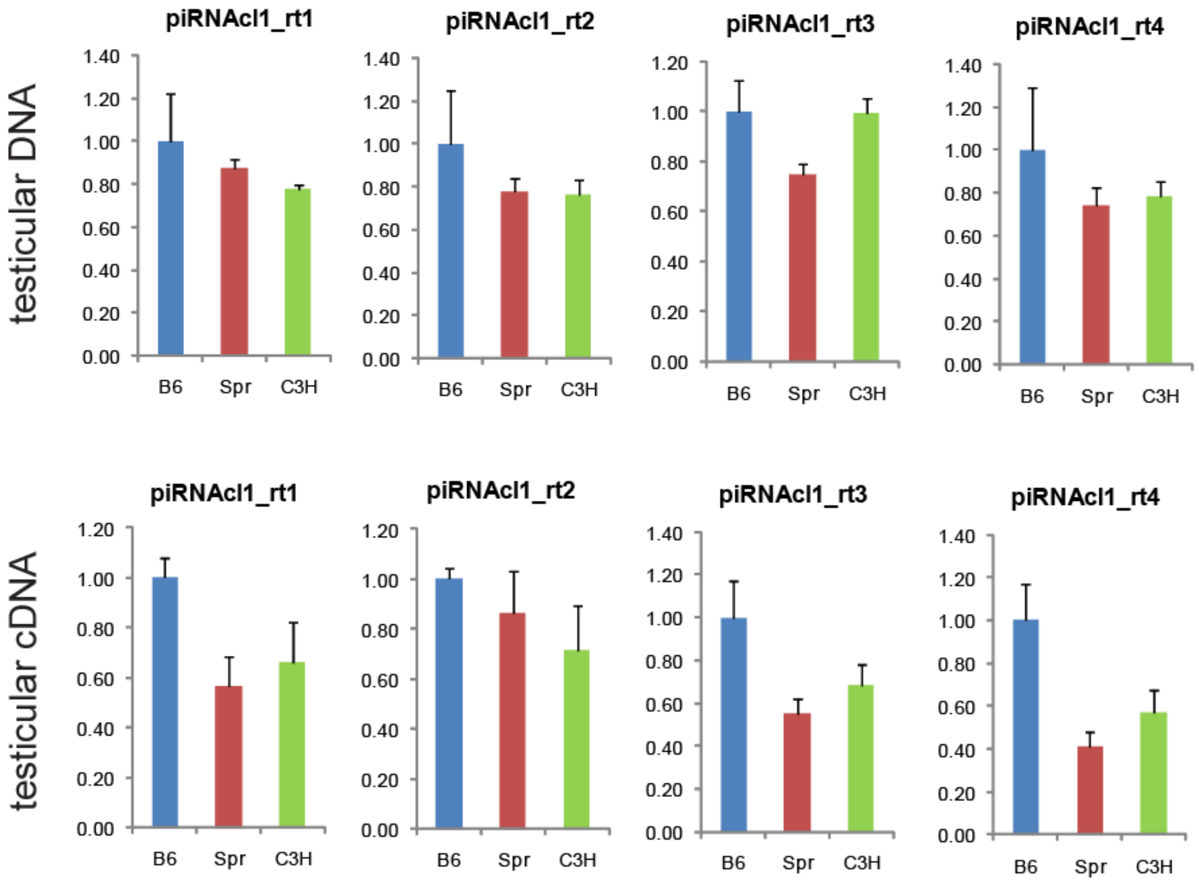

Four primers were designed to amplify piRNA precursors from the chromosomal region chr2: 92376642-92452541. Quantitative real-time PCR on testicular DNA provided similar results in all B6, Spr and C3H samples. Decreased levels of the piRNA precursors are apparent in both Spr and C3H for three primers used.
