# Supplementary material for: Differential Expression of Non-Coding RNAs and Continuous Evolution of the X Chromosome in Testicular Transcriptome of Two Mouse Species
Source: PLoS One. 2011 Feb 14;6(2):e17198. doi: 10.1371/journal.pone.0017198 (PMC3038937; doi:10.1371/journal.pone.0017198)
Supplement: Figure S3 — Summary of Mus spretus upregulated clusters on chromosome 2. (PDF) [file pone.0017198.s005.pdf]

Figure S3:

chromosome 2: upregulated cluster 1:

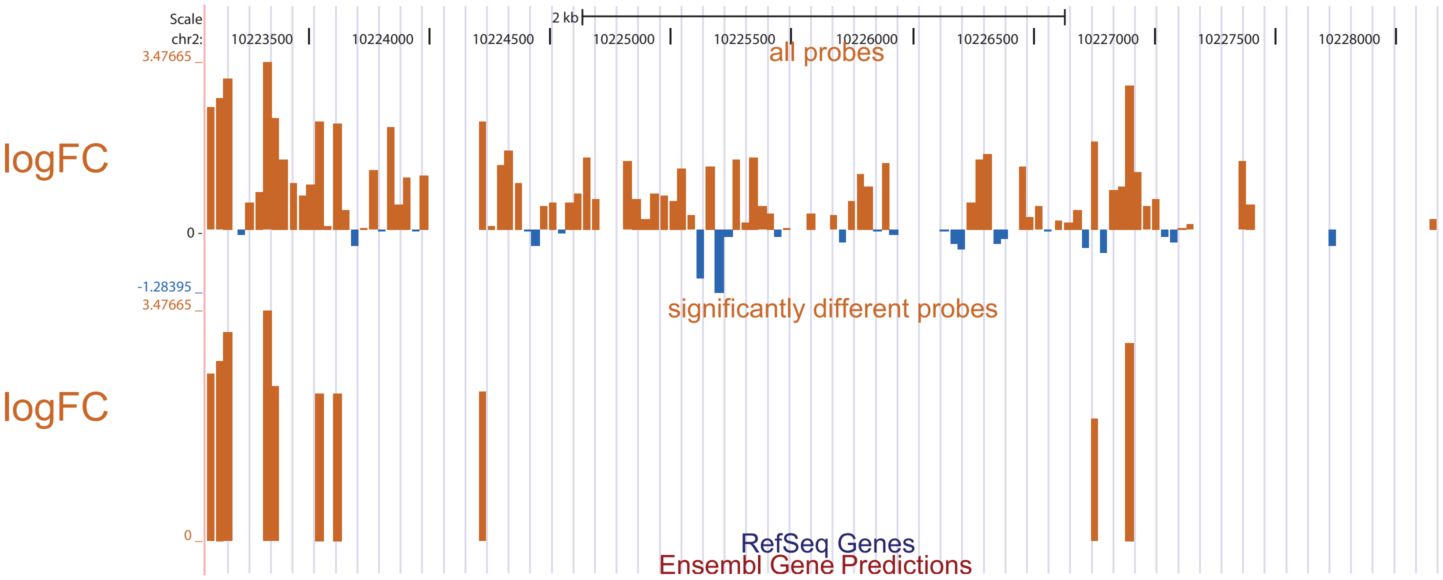

chromosome 2: upregulated cluster 2:

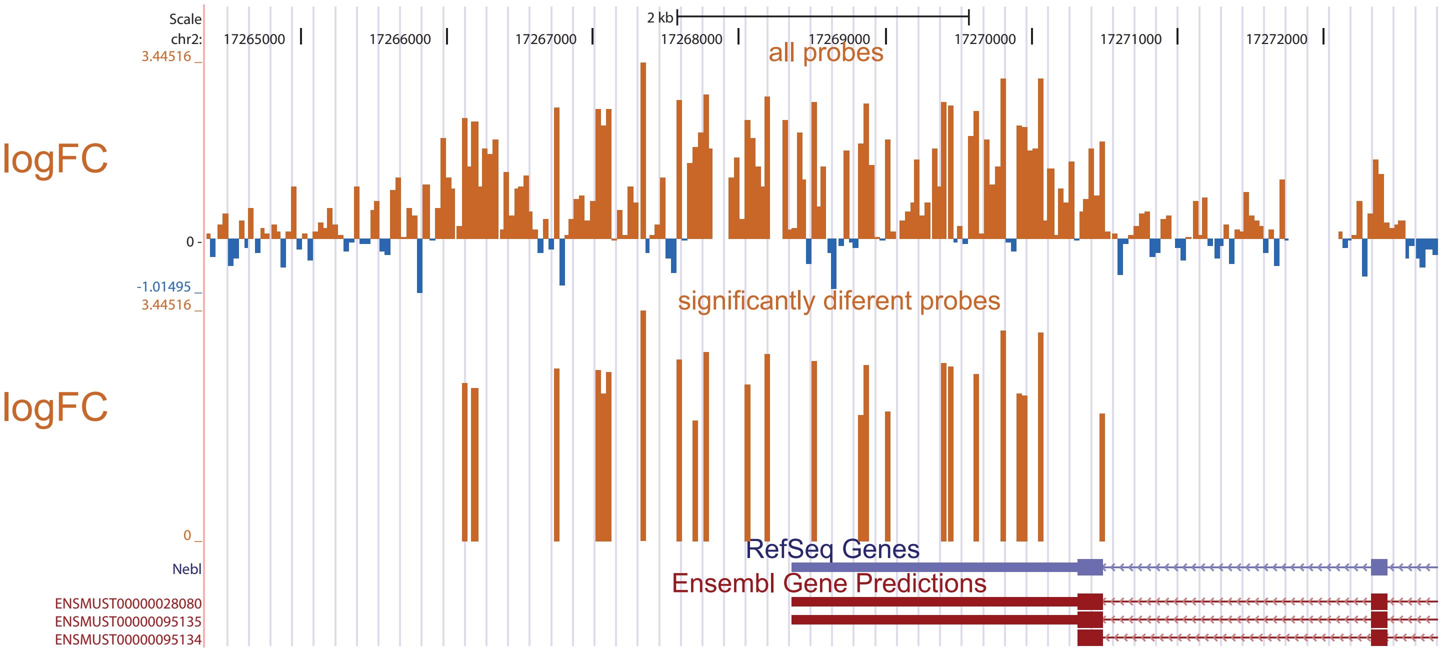

chromosome 2: upregulated cluster 3:

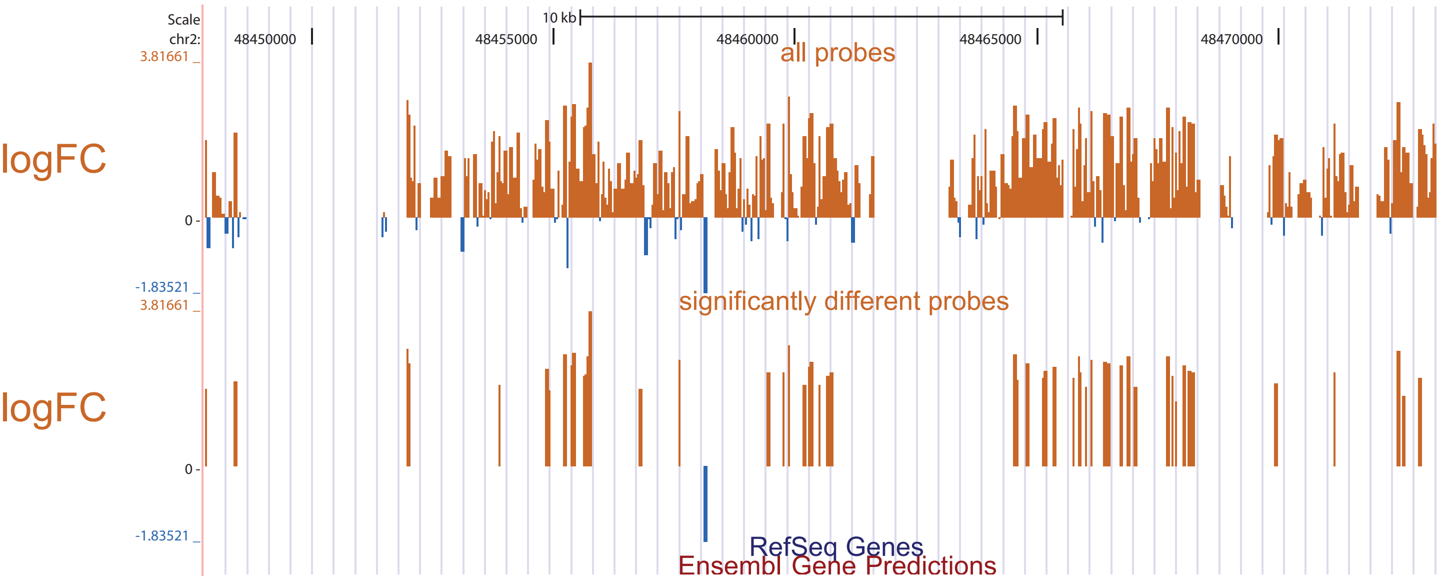

chromosome 2: upregulated cluster 4:

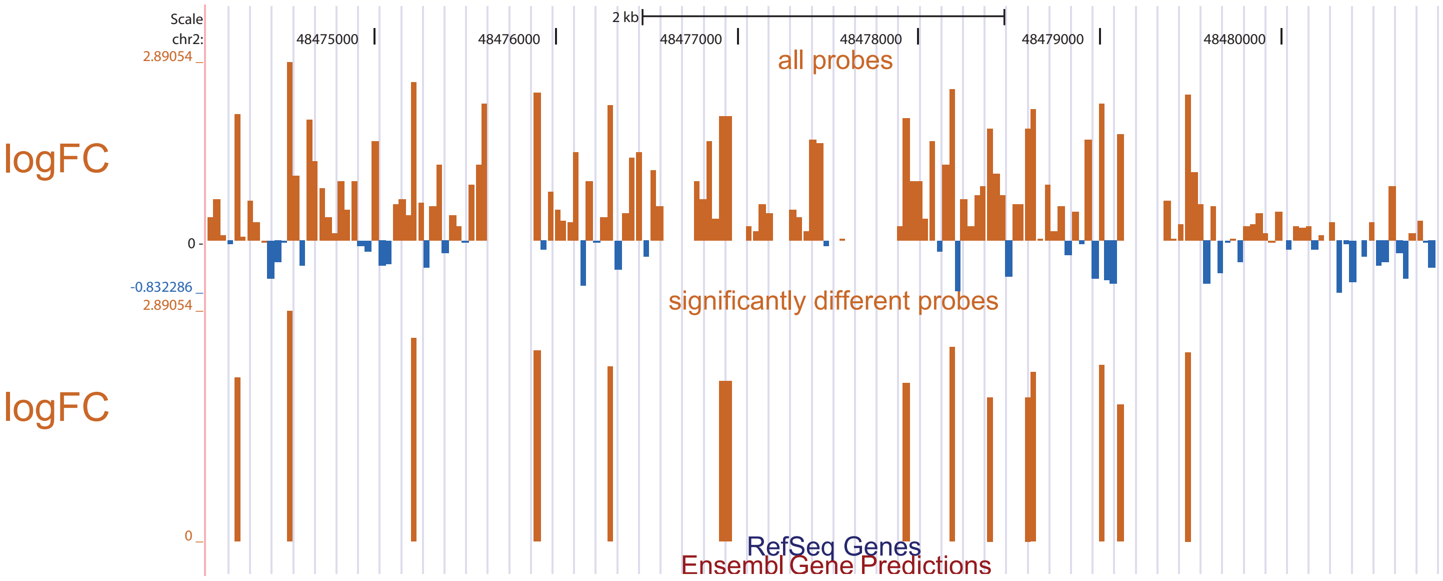

chromosome 2: upregulated cluster 5:

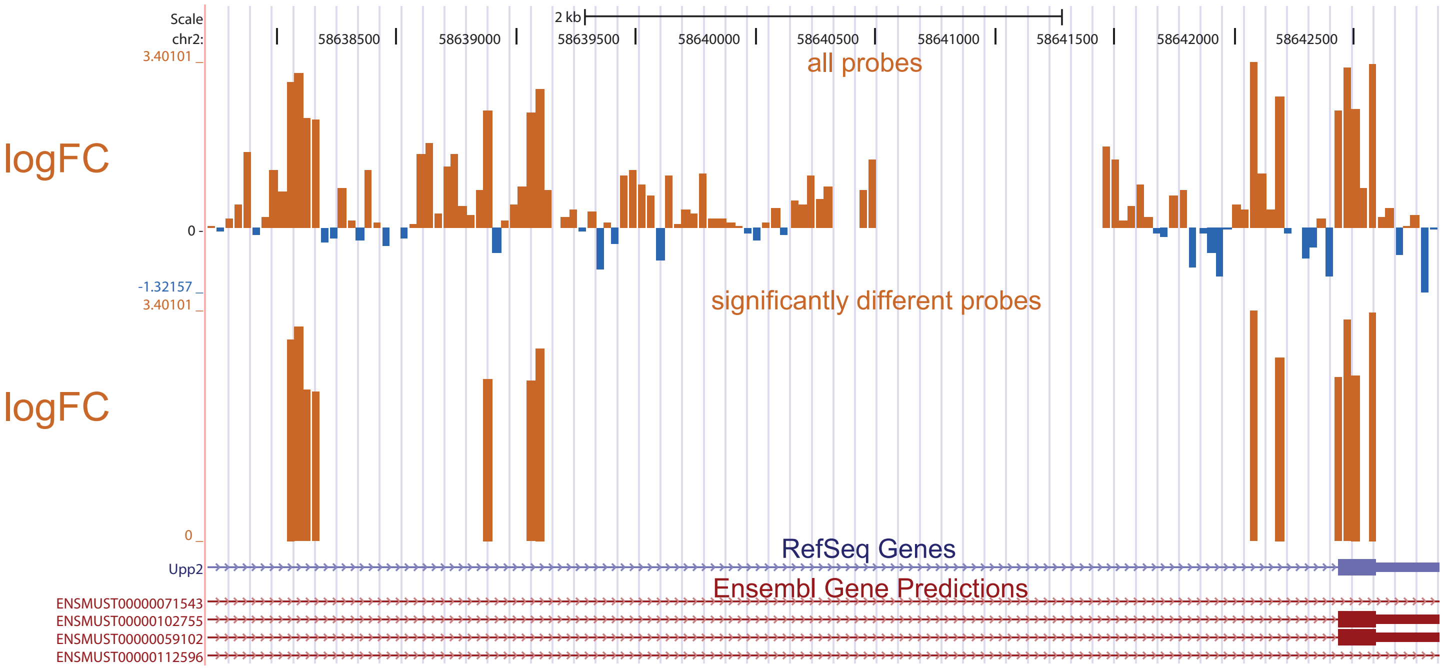

chromosome 2: upregulated cluster 6:

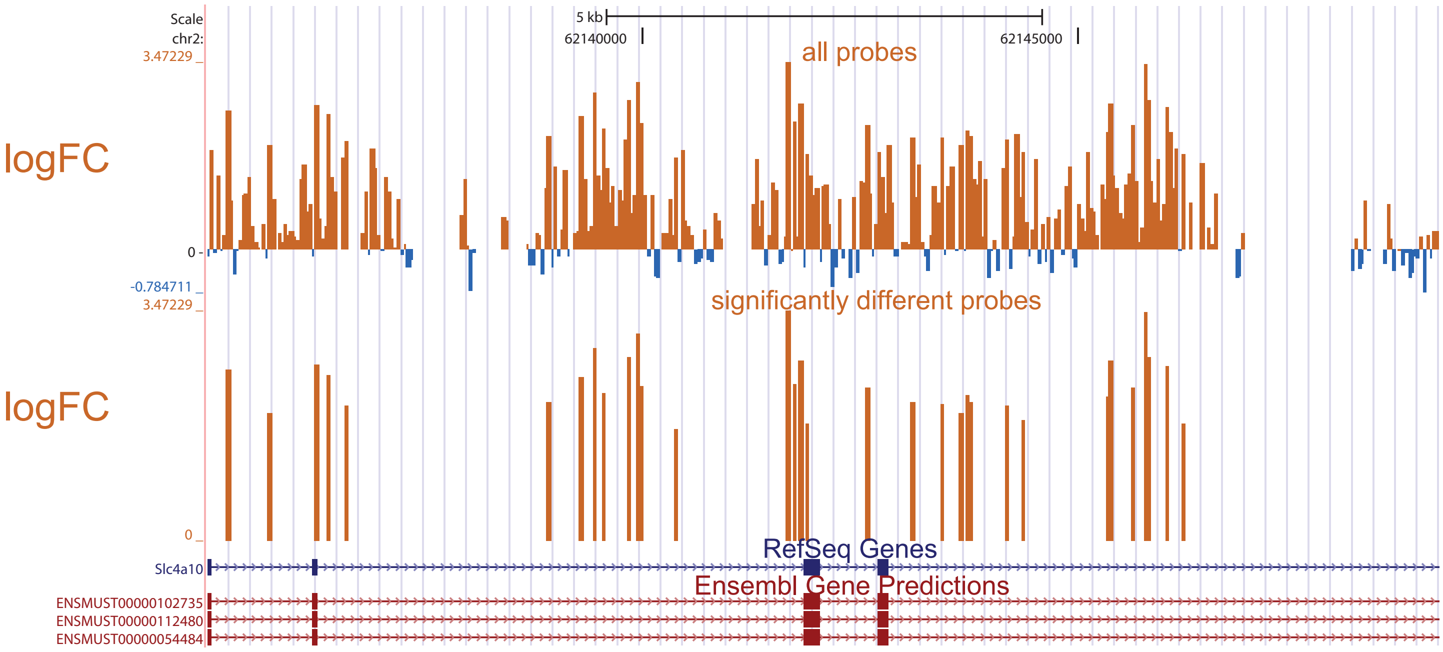

chromosome 2: upregulated cluster 7:

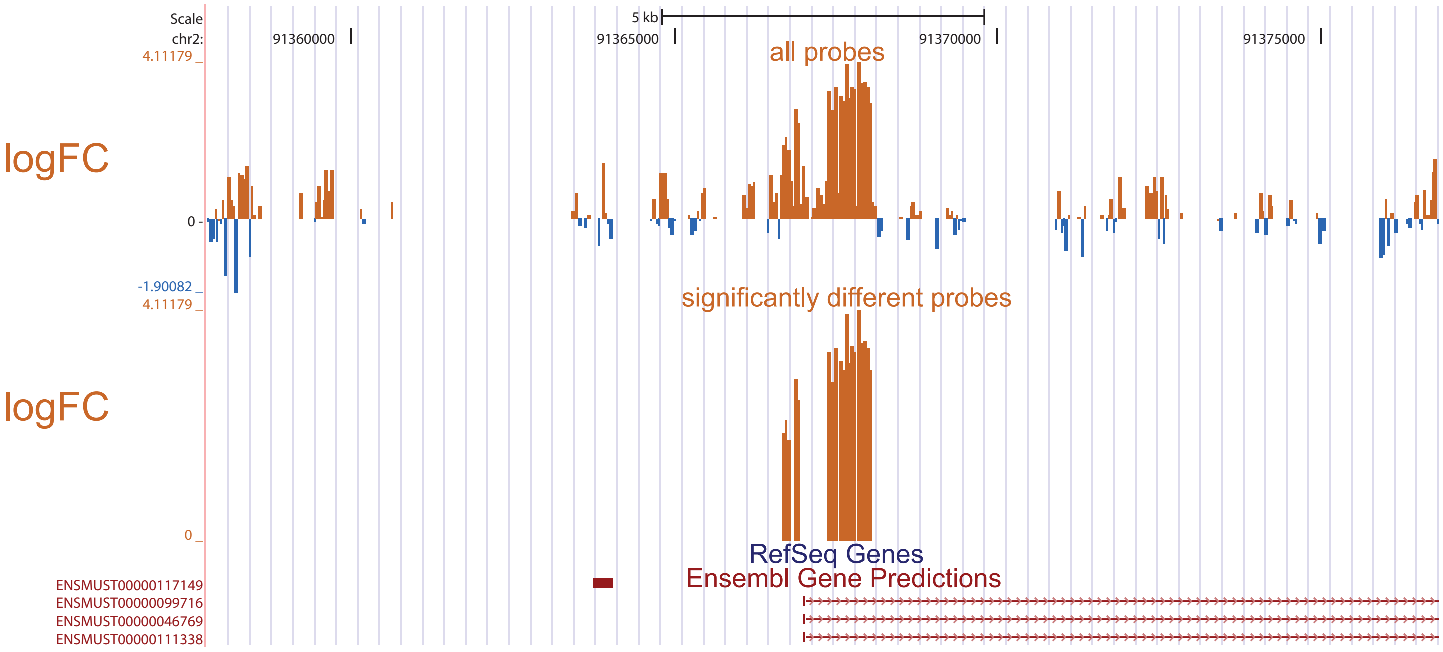

chromosome 2: upregulated cluster 8:

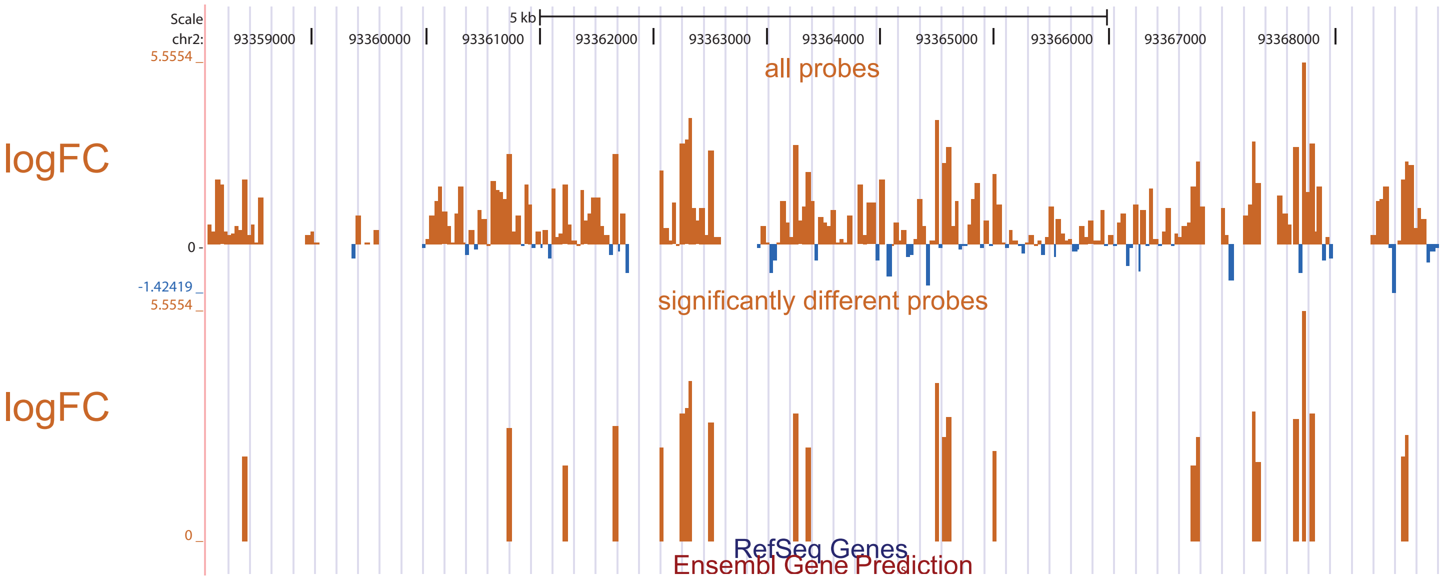

chromosome 2: upregulated cluster 9:

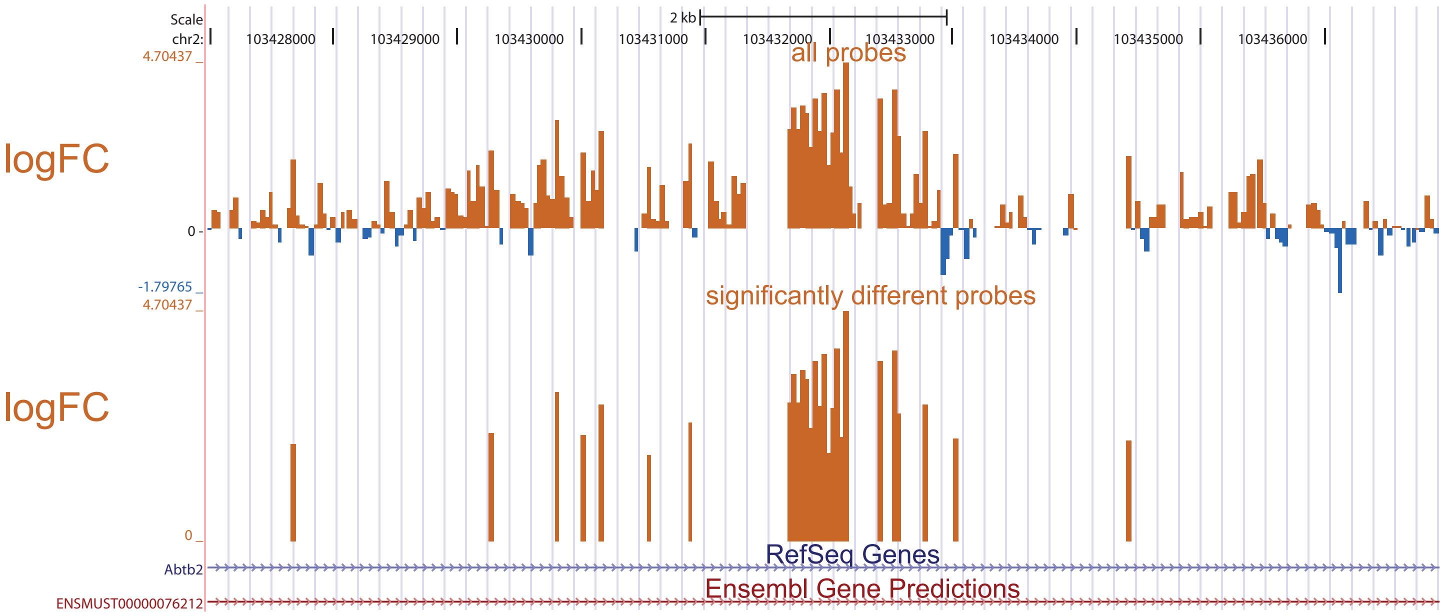

chromosome 2: upregulated cluster 10:

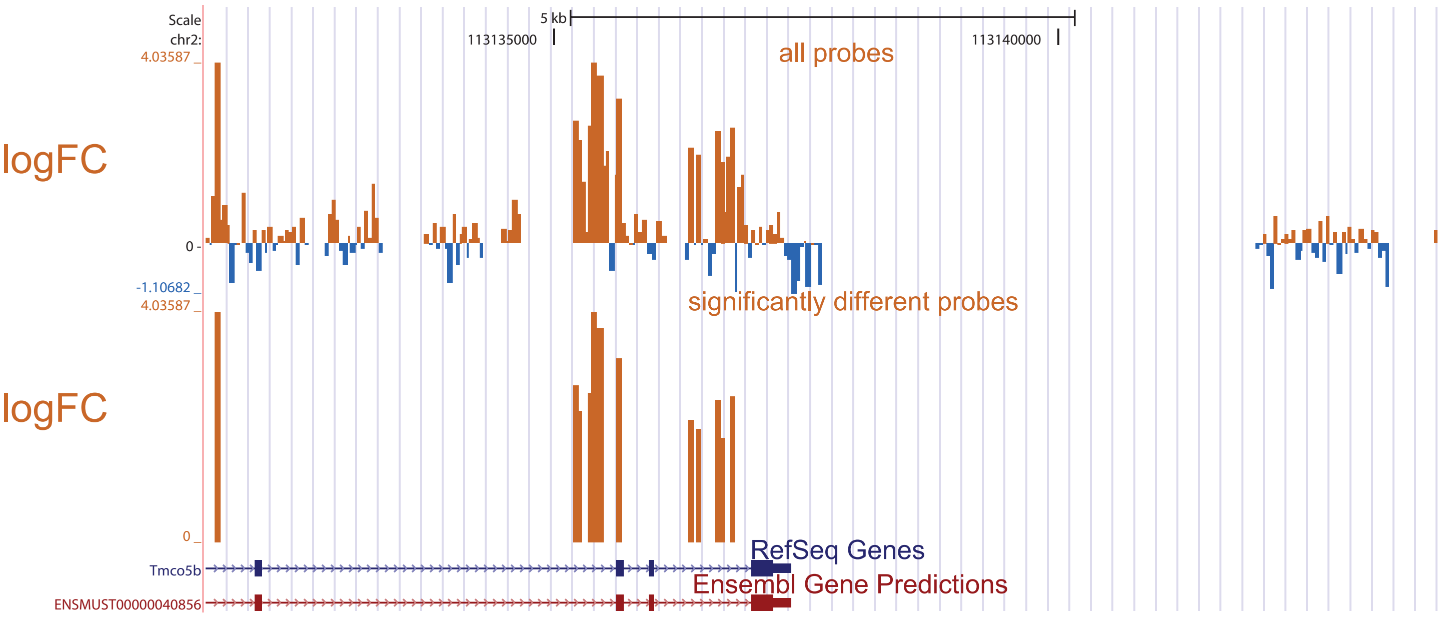

chromosome 2: upregulated cluster 11:

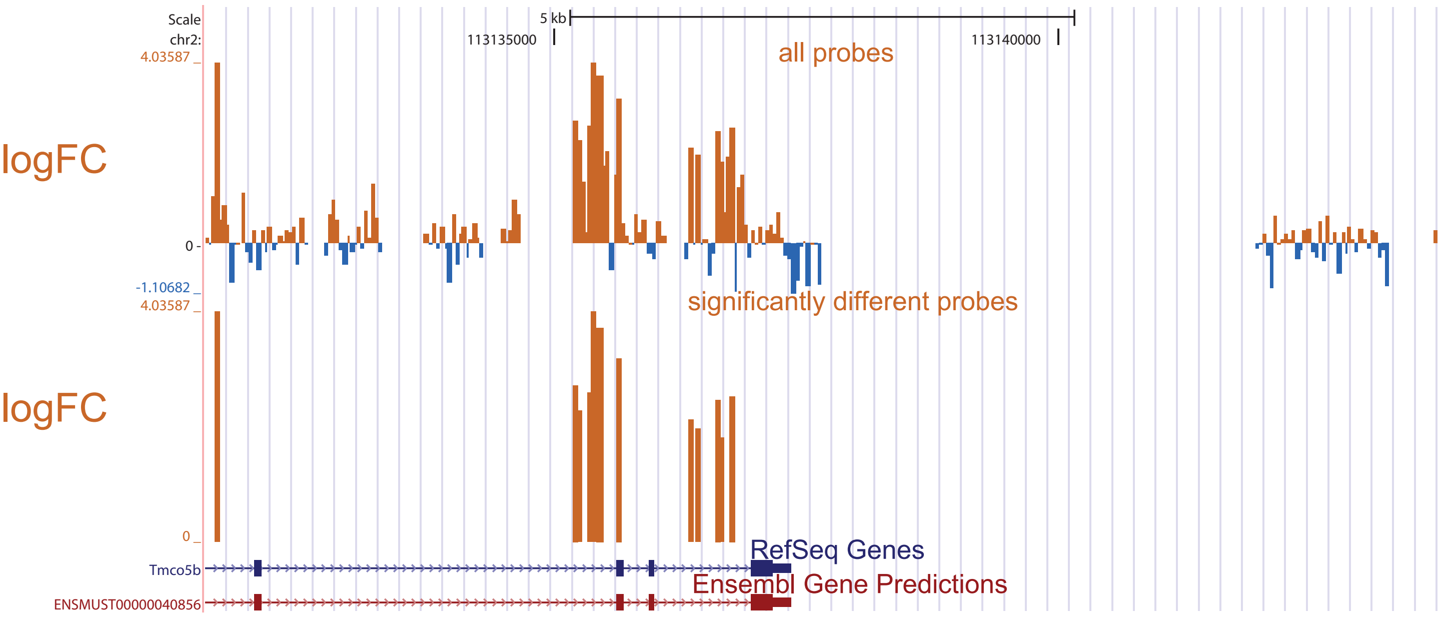

chromosome 2: upregulated cluster 12:

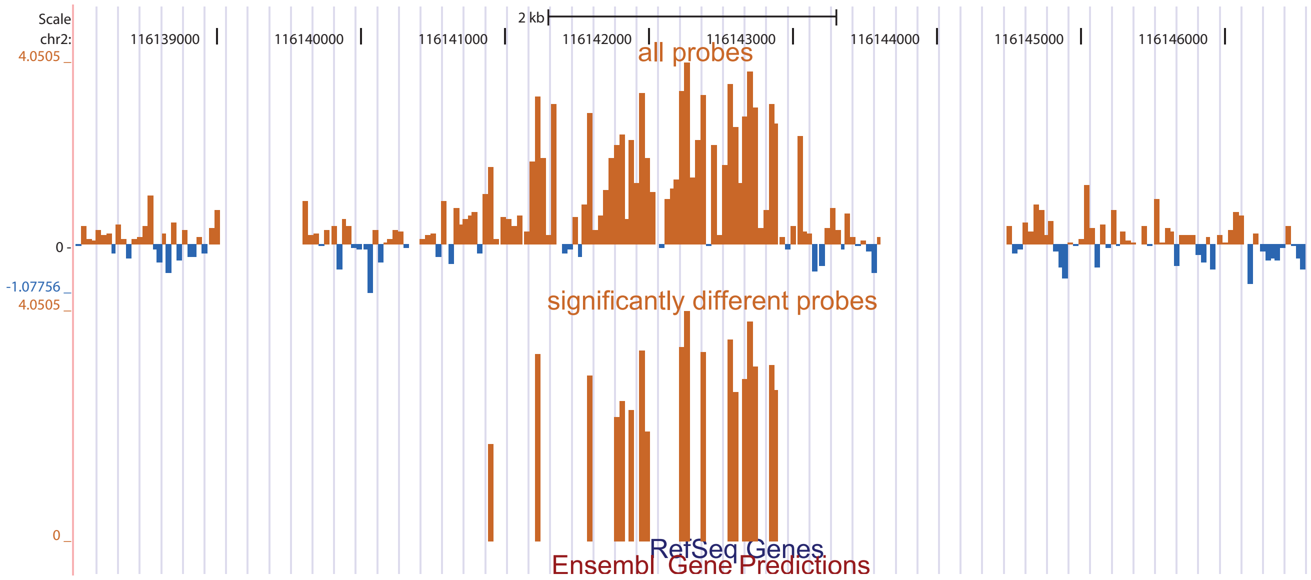

chromosome 2: upregulated cluster 13:

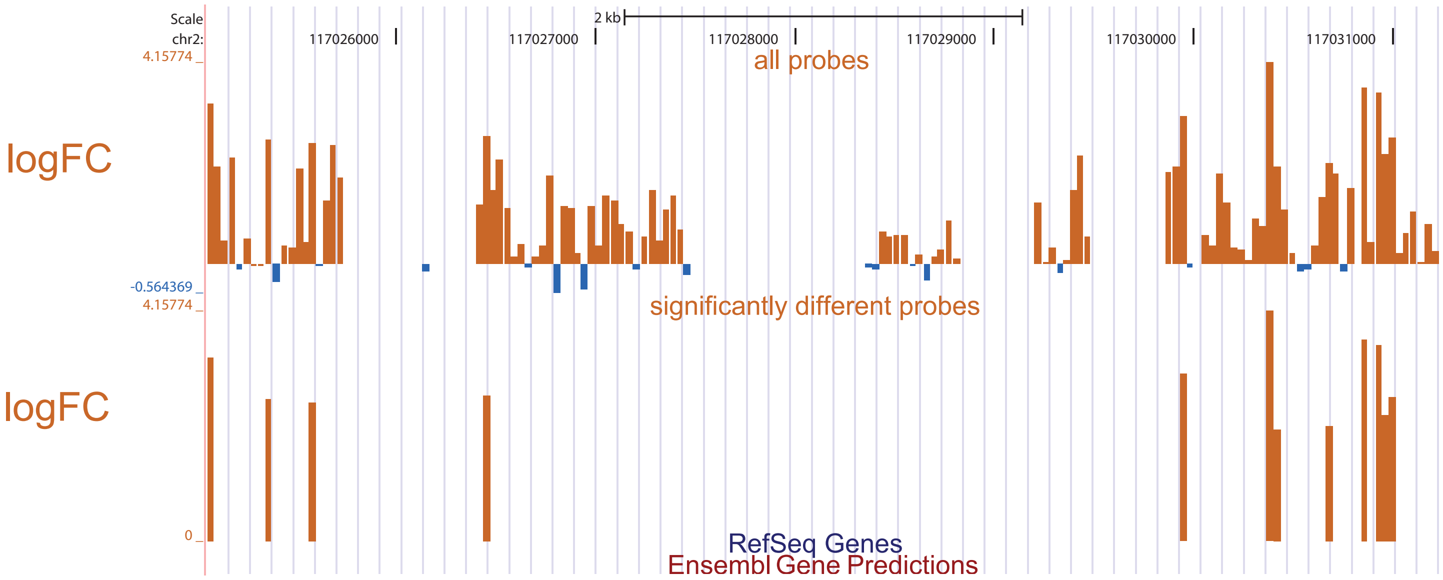

chromosome 2: upregulated cluster 14:

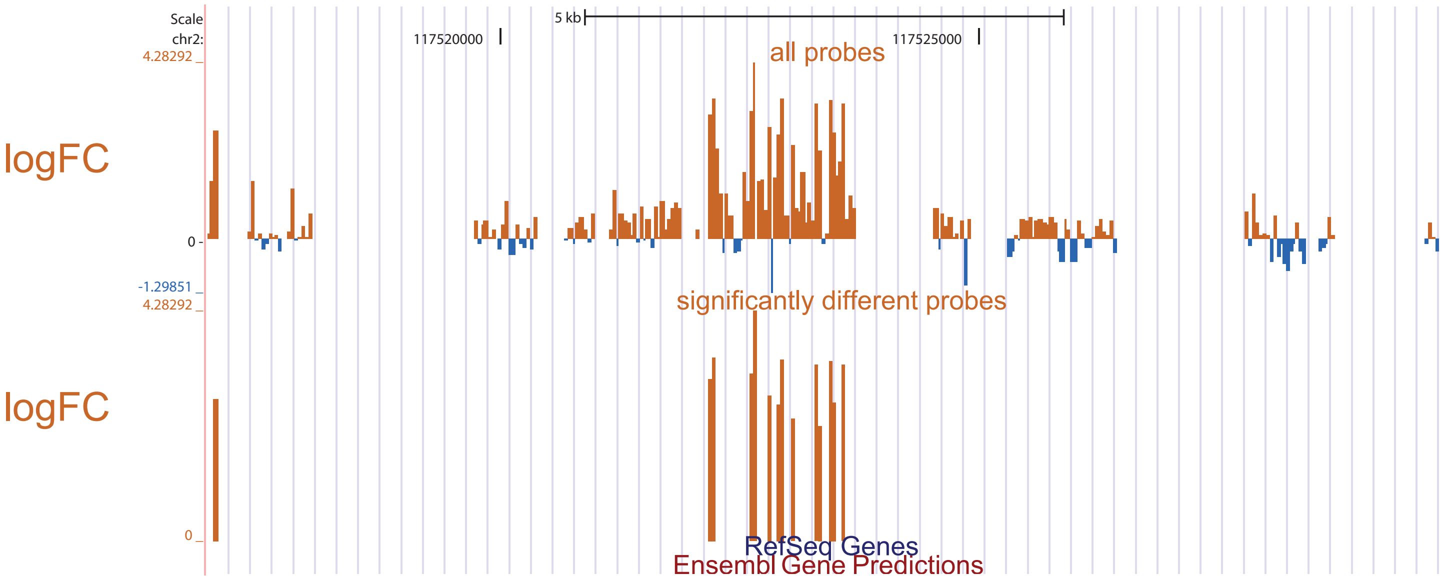

chromosome 2: upregulated cluster 15:

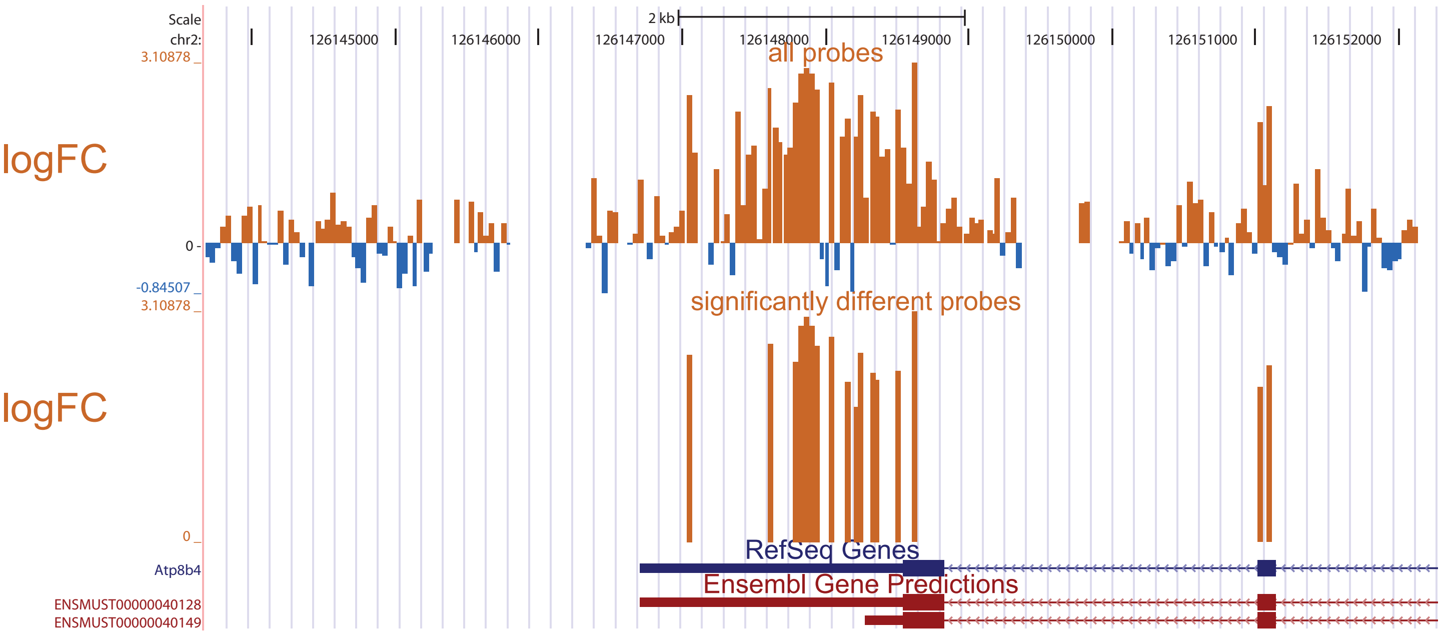

chromosome 2: upregulated cluster 16:

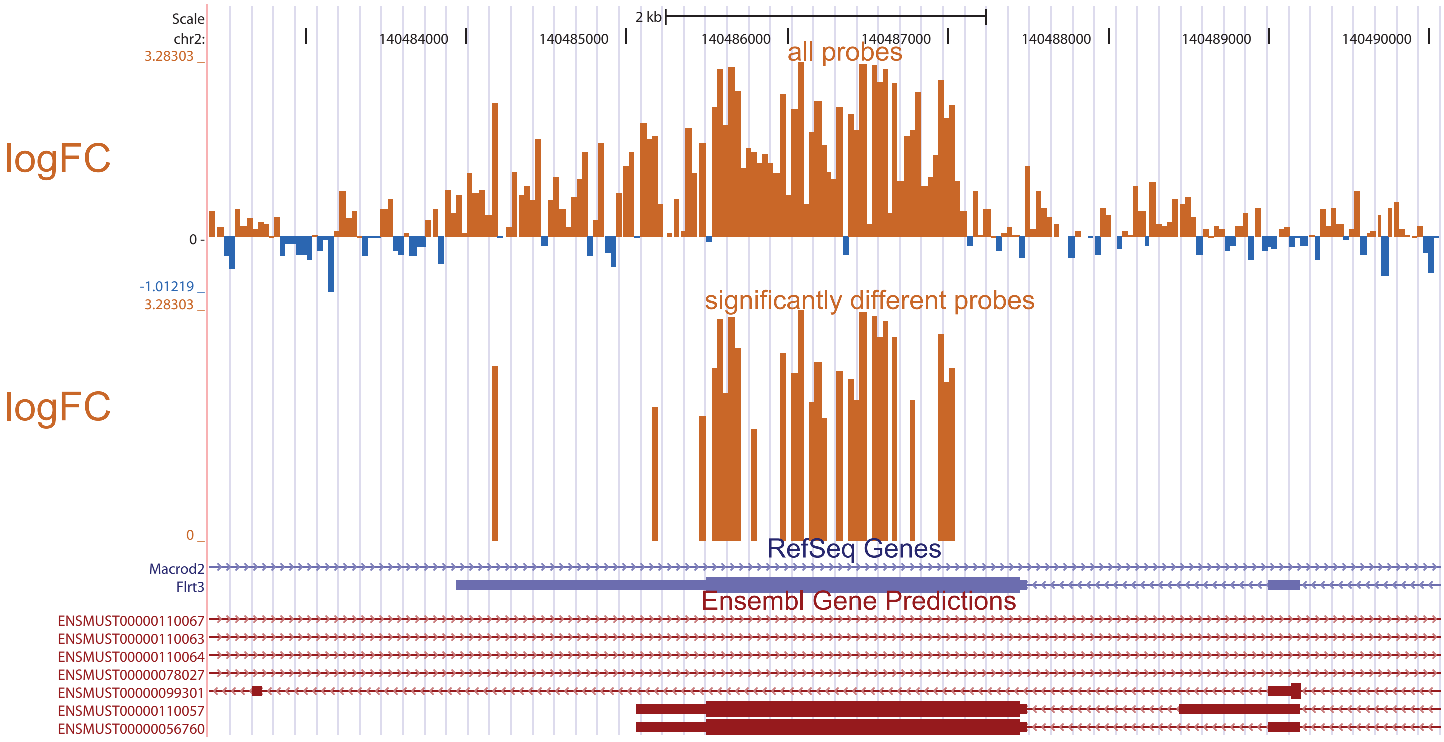

chromosome 2: upregulated cluster 17:

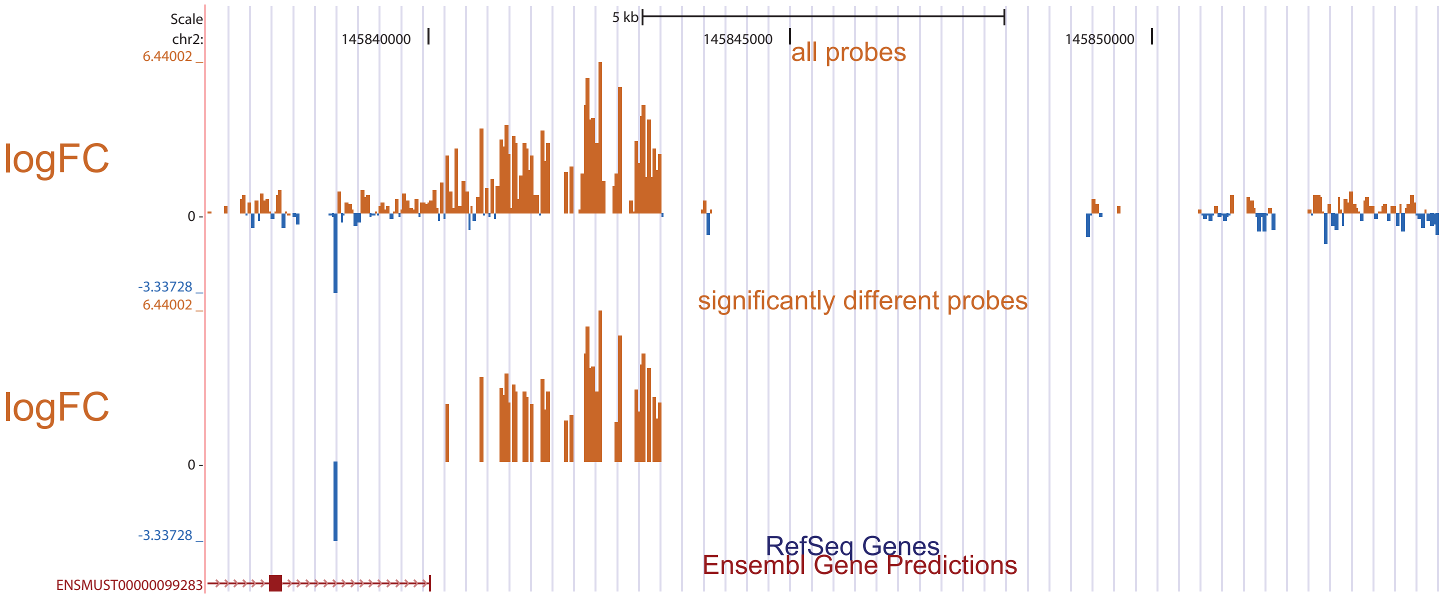

Figure S3. ***Mus spretus*** **upregulated clusters on chromosome 2**
Seventeen upregulated clusters were found on chromosome 2. Positions of the individual probes are plotted along the chromosome coordinates as bars with the height corresponding to the log fold change difference in intensity between Spr and B6. The upper track shows all the probes. The lower track displays only probes with significantly different intensity between Spr and B6 (P<0.05). Tracks with RefSeq genes and Ensembl gene predictions are also shown.
