# Supplementary material for: Differential Expression of Non-Coding RNAs and Continuous Evolution of the X Chromosome in Testicular Transcriptome of Two Mouse Species
Source: PLoS One. 2011 Feb 14;6(2):e17198. doi: 10.1371/journal.pone.0017198 (PMC3038937; doi:10.1371/journal.pone.0017198)
Supplement: Figure S4 — Copy number evaluation of G6pdx in Spretus by quantitative real-time PCR. (PDF) [file pone.0017198.s006.pdf]

Figure S4

Copy number evaluation of *G6pdx* in Spretus by quantitative real-time PCR

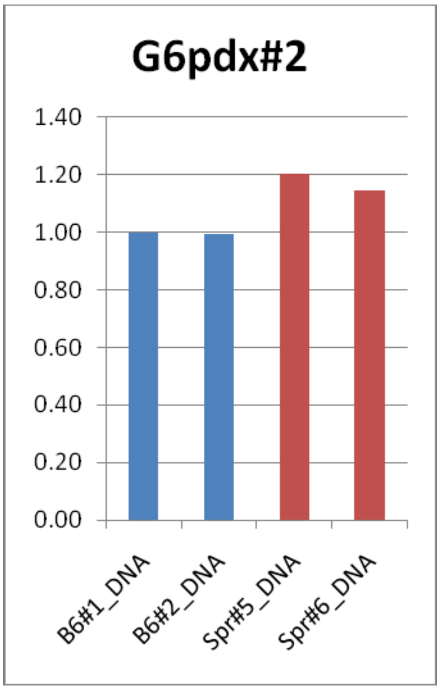

Quantitative real-time PCR (q-PCR) was performed on testicular genomic DNA from two B6 males and two Spr males using G6pdx#2 primers. Amplification of Spr and B6 genomic DNA samples achieved similar quantities as shown on the histogram. This demonstrates that *G6pdx* in Spr is a single copy gene.

The size of the amplicon refers to *G6pdx* gene

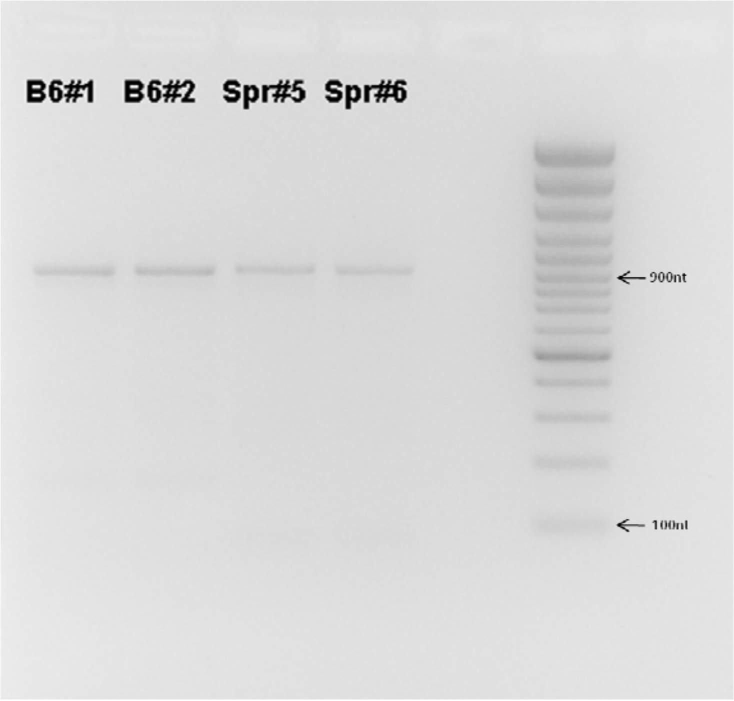

Products of q-PCR were electrophoresed on 1% agarose gel. Both B6 and Spr samples contain only PCR products of size ~907 nt, which refers to the length of genomic *G6pdx* amplicon including an intron 56?. No products of different size, especially an amplicon of 81nt size corresponding to retroposed intronless copy of the *G6pdx* gene, were detected. Thereby we proved that the G6pdx#2 primers are specific for the *G6pdx* gene and do not amplify its retrogene(s).

DNA Sequence of the amplicons refer to *G6pdx* gene in both Spr and B6

Products of q-PCR , obtained from B6#1, Spr#5 and Spr#6 genomic DNA, were sequenced using G6pdx#2 forward and reverse primers. Partial alignment of these sequences comprising the intron of *G6pdx* gene is shown together with the corresponding reference genomic sequence (UCSC build mm9: chrX: 71659673-71660032).

```
B6#1_F          AGTTGTGTCTACTTAATTGTGCCACATTCTTCCTGACCTTTGAACAAAGACAGGAAGAAG
B6#1_R          -------------TAATTGTGCCACATTCTTCCTGACCTTTGAACAAAGACAGGAAGAAG
reference       AGTTGTGTCTACTTAATTGTGCCACATTCTTCCTGACCTTTGAACAAAGACAGGAAGAAG
Spr#5_F         --------------AATTGTGCCACCTTCTTCCTGACCTTTGAACAAAGACAGGAAGAAG
Spr#5_R         AGTTGTGTCTACTTAATTGTGCCACCTTCTTCCTGACCTTTGAACAAAGACAGGAAGAAG
Spr#6_R         ------------------------------------------------------------
Spr#6_F         AGTTGTGTCTACTTAATTGTGCCACCTTCTTCCTGACCTTTGAACAAAGACAGGAAGAAG


B6#1_F          GTCCCTTCAACCTCATATCTGTCTCCTAGCTCCACTCTGGCTTCATCCTGCTATGCTCTG
B6#1_R          GTCCCTTCAACCTCATATCTGTCTCCTAGCTCCACTCTGGCTTCATCCTGCTATGCTCTG
reference       GTCCCTTCAACCTCATATCTGTCTCCTAGCTCCACTCTGGCTTCATCCTGCTATGCTCTG
Spr#5_F         GTCCCTTCAGCCTCATATCTGTCTCCTAGCTCCACTCTGGCTTCATCCTGCTATGCTCTG
Spr#5_R         GTCCCTTCAGCCTCATATCTGTCTCCTAGCTCCACTCTGGCTTCATCCTGCTATGCTCTG
Spr#6_R         --CCCTTCAGCCTCATATCTGTCTCCTAGCTCCACTCTGGCTTCATCCTGCTATGCTCTG
Spr#6_F         GTCCCTTCAGCCTCATATCTGTCTCCTAGCTCCACTCTGGCTTCATCCTGCTATGCTCTG
                  ******* **************************************************

B6#1_F          GTACCACACTGGATCATGCCAGTAGCTGGATGAAAGGACCCTTCCAAGTACCTCTTTTCT
B6#1_R          GTACCACACTGGATCATGCCAGTAGCTGGATGAAAGGACCCTTCCAAGTACCTCTTTTCT
reference       GTACCACACTGGATCATGCCAGTAGCTGGATGAAAGGACCCTTCCAAGTACCTCTTTTCT
Spr#5_F         GTACCACACTGGATCATGCCAGTAGCTGGATGAAAGGACCCTTCCAAGTACCTCTTTTCT
Spr#5_R         GTACCACACTGGATCATGCCAGTAGCTGGATGAAAGGACCCTTCCAAGTACCTCTTTTCT
Spr#6_R         GTACCACACTGGATCATGCCAGTAGCTGGATGAAAGGACCCTTCCAAGTACCTCTTTTCT
Spr#6_F         GTACCACACTGGATCATGCCAGTAGCTGGATGAAAGGACCCTTCCAAGTACCTCTTTTCT
                ************************************************************

B6#1_F          ACAGTAATTGCACTATGTTGACATTTTCTCACCACCTATAGTTTACTGTAGTAATTTTGA
B6#1_R          ACAGTAATTGCACTATGTTGACATTTTCTCACCACCTATAGTTTACTGTAGTAATTTTGA
reference       ACAGTAATTGCACTATGTTGACATTTTCTCACCACCTATAGTTTACTGTAGTAATTTTGA
Spr#5_F         ACAGTAATTGCACTATGTTGACATTTTCTCTCCACCTATAGTTTACTGTGGTAATTTTAG
Spr#5_R         ACAGTAATTGCACTATGTTGACATTTTCTCTCCACCTATAGTTTACTGTGGTAATTTTAG
Spr#6_R         ACAGTAATTGCACTATGTTGACATTTTCTCTCCACCTATAGTTTACTGTGGTAATTTTAG
Spr#6_F         ACAGTAATTGCACTATGTTGACATTTTCTCTCCACCTATAGTTTACTGTGGTAATTTTAG
                ****************************** ****************** ********

B6#1_F          AGAGACAACCTTCTCCATCATTCTTTTTTCTCAGGTTAGACTTTCCTGAAATCCAGTTTC
B6#1_R          AGAGACAACCTTCTCCATCATTCTTTTTTCTCAGGTTAGACTTTCCTGAAATCCAGTTTC
reference       AGAGACAACCTTCTCCATCATTCTTTTTTCTCAGGTTAGACTTTCCTGAAATCCAGTTTC
Spr#5_F         AGAGACAACCTTCTCCATCATTCATTTTTCTCAGGTTAGACTTTCCTGAAATCCAGTTTC
Spr#5_R         AGAGACAACCTTCTCCATCATTCATTTTTCTCAGGTTAGACTTTCCTGAAATCCAGTTTC
Spr#6_R         AGAGACAACCTTCTCCATCATTCATTTTTCTCAGGTTAGACTTTCCTGAAATCCAGTTTC
Spr#6_F         AGAGACAACCTTCTCCATCATTCATTTTTCTCAGGTTAGACTTTCCTGAAATCCAGTTTC
                *********************** ************************************

B6#1_F          TACTCTAGGTATGAGGGCCCCAAGGATACATAAAAATGTGCCAGTGATTTGCCCCACCTG
B6#1_R          TACTCTAGGTATGAGGGCCCCAAGGATACATAAAAATGTGCCAGTGATTTGCCCCACCTG
reference       TACTCTAGGTATGAGGGCCCCAAGGATACATAAAAATGTGCCAGTGATTTGCCCCACCTG
Spr#5_F         TACTCTAGGTATGAGGGCCCCAAGGATACAT-----------------------------
Spr#5_R         TACTCTAGGTATGAGGGCCCCAAGGATACATAAAAATGTGCCAGTGATTTGCCCCACCTG
Spr#6_R         TACTCTAGGTATGAGGGCCCCAAGGATACATAAAAATGTGCCAGTGATTTGCCCCACCTG
Spr#6_F         TACTCTAGGTATGAGGGCCCCAAGGATACATAAAAATGTGCCAGTGATTTGCCCCACCTG
                *******************************
```
